# Contribution of the Cpx envelope stress system to metabolism and virulence regulation in *Salmonella enterica* serovar Typhimurium

**DOI:** 10.1371/journal.pone.0211584

**Published:** 2019-02-04

**Authors:** Sivaraman Subramaniam, Volker S. Müller, Nina A. Hering, Hans Mollenkopf, Daniel Becker, Ann Kathrin Heroven, Petra Dersch, Anne Pohlmann, Karsten Tedin, Steffen Porwollik, Michael McClelland, Thomas F. Meyer, Sabine Hunke

**Affiliations:** 1 Department of Microbial Physiology, Humboldt Universität zu Berlin, Berlin, Germany; 2 Genomic Core Facility, Max-Planck-Institute for Infection Biology, Berlin, Germany; 3 Department of Molecular Biology, Max-Planck-Institute for Infection Biology, Berlin, Germany; 4 Department of Infection Biology, Helmholtz Center for Infection Research, Braunschweig, Germany; 5 Department of Microbiology, Humboldt Universität zu Berlin, Berlin, Germany; 6 Centre for Infection Medicine, Institute of Microbiology and Epizootics, Freie Universität Berlin, Berlin, Germany; 7 Department of Microbiology and Molecular Genetics, University of California, Irvine, California, United States of America; Institut National de la Recherche Agronomique, FRANCE

## Abstract

The Cpx-envelope stress system regulates the expression of virulence factors in many Gram-negative pathogens. In *Salmonella enterica* serovar Typhimurium deletion of the sensor kinase CpxA but not of the response regulator CpxR results in the down regulation of the key regulator for invasion, HilA encoded by the *Salmonella* pathogenicity island 1 (SPI-1). Here, we provide evidence that *cpxA* deletion interferes with dephosphorylation of CpxR resulting in increased levels of active CpxR and consequently in misregulation of target genes. 14 potential operons were identified to be under direct control of CpxR. These include the virulence determinants ecotin, the omptin PgtE, and the SPI-2 regulator SsrB. The Tat-system and the PocR regulator that together promote anaerobic respiration of tetrathionate on 1,2-propanediol are also under direct CpxR control. Notably, 1,2-propanediol represses *hilA* expression. Thus, our work demonstrates for the first time the involvement of the Cpx system in a complex network mediating metabolism and virulence function.

## Introduction

An important group of bacterial regulatory sensing systems are the two-component systems, each of which enable bacteria to sense and respond to a specific subset of environmental changes and stress factors [1–3]. Two-component systems recognize environmental changes via a membrane-anchored sensor kinase that mediates the response through phosphorylation and dephosphorylation of its cognate response regulator [1]. The phosphorylated response regulator modulates the expression of target genes [3]. The Cpx-envelope stress system is a two-component system ubiquitous among Gram-negative pathogens [4, 5]. It is composed of the sensor kinase CpxA, the response regulator CpxR and the auxiliary periplasmic protein CpxP that inhibits CpxA presumably through a direct dynamic interaction [6, 7]. The Cpx-system corresponds to signals that induce envelope stress such as elevated pH, increased osmolarity, indole, adrenalin, surface contact and accumulation of adhesin subunits [5, 8–12]. Activation of the Cpx-system results in CpxA autophosphorylation and subsequently the phosphoryl group transferred to CpxR [6, 13]. Interestingly, all these signals typically emerge during early stages of infection in the gut and, accordingly, the Cpx-system could be linked to the virulence of enteropathogenic *Escherichia*, *Salmonella*, *Shigella*, *Vibrio* and *Yersinia* species [4, 5, 14–16]. A deletion of the *Salmonella* Cpx-system showed significantly reduced abilities to colonize tissue and inner organs in pigs [17, 18].

Several studies demonstrated the impact of the Cpx-system for *Salmonella enterica* virulence. In *Salmonella enterica* serovar *Typhi* (*S*. Typhi), the causative agent of human typhoid fever, inactivation of CpxA results in a mutant defective for adherence and invasion of human small intestinal epithelial cell lines [19]. Thereby, the expression of the *cpxA* is regulated under conditions of high osmolarity (0.3 M NaCl) and is pH independent [19]. Inactivation of CpxA in *Salmonella enterica* serovar *Typhimurium* (*S*. *typhimurium*), a causative agent of gastroenteritis in humans and a typhoid-like fever in mice, leads to a strain defective in both invasion and transcription of the regulator for invasion HilA when grown under mild acid condition [20]. In contrast, deletion of *cpxR* and growth under neutral or mild alkaline pH has no effect [20, 21]. Moreover, a *cpxA* mutant, but not a *cpxR* mutant, was slightly attenuated in mice virulence after oral and parenteral infection [21]. From these cumulative results it was suggested that CpxA might modulate the expression of HilA and consequently invasion independently of its cognate response regulator CpxR, presumably through cross-talk with another regulator [5, 20].

In addition to invasion, the Cpx-system is involved in resistance to cationic antimicrobial peptides (CAMPs) [22]. Two N-acetylmuramoyl-L-alanin amidases, encoded by the genes amiA and amiC, are direct CpxR targets in S. Typhimurium [22]. These amidases are secreted by the twin arginine translocation [23] system to the periplasmic space and contribute to bacterial resistance to the CAMPs protamine, magainin 2 and melittin but do not contribute to resistance tothe CAMPs HNP-1 and polymyxin B [22]. The authors confirmed the impact of the Cpx-system on the resistance to CAMPs for protamine and magainin 2 and melittin and suggested that the Cpx-system contributes resistance to protamine in a Tat-independent way [22]. The Cpx system has been demonstrated to be implicated in neuroendocrine hormone-mediated haemolysis in *S*. Typhi, indicating the importance of the Cpx system for pathogen-host cross-talk [10].

A comprehensive analysis for the impact of the Cpx-system for the virulence of the model *S*. Typhimurium SL1344 has been performed using inactivation and constitutive activation strategies involving host interaction model [21]. However, a global analysis involving a genome wide approach to identify *S*. Typhimurium specific CpxR targets that might contribute to virulence is missing. Here, we used a combination of global transcriptional and bioinformatic analysis to uncover previously uncharacterized members of the CpxR regulatory network. We identified 14 potential novel CpxR target genes and demonstrated that CpxR not only directly controls the transcription of the serine protease inhibitor ecotin, the omptin PgtE, the SPI-2 regulator SsrB and the Tat-system but also has an unsuspected role in the metabolism of 1,2-propanediol, an abundant compound in the human intestine. Moreover, we provide evidence that *cpxA* deletion interferes with dephosphorylation of CpxR under invasion inducing conditions. We propose that in the absence of CpxA under invasion inducing conditions CpxR may be constitutively phosphorylated by another kinase or phosphodonor leading to repression of the SPI-1 regulator HilA. Accordingly, dephosporylation of CpxR by CpxA restores *hilA* expression by preventing the negative effects of active CpxR on this gene. Thus, this study suggests that both activities of CpxA—phosphorylation and dephosphorylation of CpxR are critical to control CpxR-mediated virulence gene regulation.

## Materials and methods

### Growth media and conditions

Standard media for *Salmonella* enterica serovar Typhimurium (*S*. Typhimurium) and *E*. *coli* was lysogeny broth [21], in the presence of appropriate antibiotics. Minimal medium was the No-carbon-E (NCE) medium, supplemented with trace metals (0.3 mM CaCl2, 0.1 mM ZnSO4, 0.045 mM FeSO4, 0.2 mM Na2Se2O3, 0.2 mM Na2MoO4, 2 mM MnSO4, 0.1 mM CuSO4, 3 mM CoCl2, and 0.1 mM NiSO4) and 0.2% yeast extract as carbon source with or without 80 mM propanediol (Sigma) [24]. The antibiotics used for bacterial selection on plates, cultures were as follows: ampicillin 100 μg ml-1, kanamycin 50 μg ml-1, streptomycin 90 μg ml-1 and chloramphenicol 20 μg ml-1. Salmonella invasion inducing media contained 0.3 M NaCl in LB pH 7.0. For motility assays bacteria were precultured in Terrific Broth (TB).

### Strain and plasmid construction

Bacterial strains and plasmids used in this study are listed in Table 1. *E*. *coli* strain JM109 was used for cloning and *E*. *coli* strain BL21DE3 for protein expression. SL1344 is a standard virulent strain of *S*. Typhimurium. The *cpxA*, *cpxR* and *cpxRA* deletion mutants were constructed in *S*. Typhimurium LT2 as described [25–27], confirmed by PCR and P22 transduced into the parent SL1344.

**Table 1:** *E*.*coli* strains and plasmids used in this study.

**Strain / Plasmid**	**Relevant Gentotype**	**Reference or Source**
MG1655	F- lambda- *ilvG*- *rfb*-50 *rph*-1	[28]
JM109	e14^-^ (McrA^-^) *recA1 endA1 gyrA96 thi-1 hsdR17* (r_K_^-^m_K_^+^) *supE44 relA1*	Stratagene
BL21DE3	F-, *gal met r—m*- *hdsS* λlysp*la*cUV5-T7-Gen1 p*lacI*q *lacI*	[29]
SL1344	wild-type, Str^R^ *hisG rpsL xyl*	[30]
KT04	SL1344 *hisG46 rpsL fliC fljB*	Lab collection K.Tedin
NOS01	SL1344 *cpxR*::*kan*	This study
SHS01	SL1344 *cpxA*::*kan*	This study
pACYC184	cloning vector, p15A, Cam^R^	[31]
pBR322	cloning vector, Tet^R^, Amp^R^	[32]
pD2E	pGFP_OVA_, *hilA-gfp*_*ova*_, Amp^R^	[33]
pGFP_OVA_	pBR322 with GFP-OVA, Amp^R^	[34]
pIVEX2.4	T7 overexpression vector, Amp^R^	Roche
pIStmCpxR	pIVEX2.4, *cpxR*_STM_^+^, Amp^R^	This Study
pKD4		[27]
pKD13		
pKD46		[27]
pSSS11		This Study

The coding region of *cpxR* was cloned into the NcoI and BamHI sites of pIVEX2.4, resulting in pIStmCpxR. pSSS11 was achieved by cloning the *cpxRA* coding region into the BamHI and SalI sites of pACYC184. All constructed plasmids were confirmed by sequencing. DNA manipulation, restriction digestion, ligations and transformations were performed using standard genetic and molecular techniques [35].

### Measurement of gene expression

The activity of the *hilA*-GFP fusion encoded on the plasmids pD2E, was measured under SPI-1 inducing growth conditions (LB-pH7.0 with 0.3M NaCl under oxygen limiting static conditions). A fresh colony was inoculated into 5 ml SPI-1 media and grown for 2 hours (OD600 of 0.5). For fluorimeter measurements cultures were diluted 1:50 in 150 μl SPI-1 Media and transferred into Costar 96 black clear bottom plates (Corning Life Sciences; The Netherlands). Plates were sealed with adhesive sealing films (Roth, Germany) and cell growth (OD620) and GFP-production were monitored every 17 minutes over a timeline of 6 h in a Fluorimeter (BD Biosciences). The fluorescent values were measured at excitation values set at 485 nm and emission values set at 540 nm. Assays were performed with an n = 6 and normalized to the wildtype control.

### RNA isolation and labelling for DNA microarray

Total bacterial RNA was isolated from bacteria grown under different growth conditions after killing by the addition of 0.2 volumes of 95% ethanol, 5% phenol, pH 4.3. Pellets were resuspended in 10 mM Tris, 1 mM EDTA containing 2 mg ml-1 lysozyme and incubated at 37°C for 30 min. Cell lysis solution (Qiagen, Hilden, Germany) was added and the mixture was incubated at 65°C for 5 min and at room temperature for 10 min. After the addition of precipitation solution (Qiagen, Hilden, Germany) and incubation on ice for 5 min cell debris, proteins and DNA were pelleted. The RNA containing supernatant was mixed with ethanol and loaded on a spin column (Promega). Further RNA purification and DNase digestion was done as described by the manufacturer.

A total of 50 μg of RNA of six separate experiments was reverse transcribed to cDNA and labelled with Cy3- or Cy5-conjugated dCTP (GE Healthcare) using reverse transcriptase (SupersciptII, Invitrogen) and random hexamers as primers. RNA was removed by hot-alkali treatment. Labelled cDNA was purified using a Qiaquick PCR purification kit and quantified by Nano-Drop analysis (ND-1000 Spectrophotometer, Peqlab).

### DNA microarrays and data analysis

Slides containing three replicate arrays spotted onto CMT-UltraGAPS (Corning) slides were prehybridized in 25% formamide, 5 x SSC and 0.1% SDS at 42°C. Probes were prepared by mixing equal amounts of Cy3- or Cy5- labelled cDNA from wild type and the cpxA mutant strain with an equal volume of hybridization solution (50% formamide, 10 x SSC and 0.2% SDS). After hybridization and washing, arrays were scanned with a Microarray Scanner BA (Agilent Technologies) at 5 μm resolution. Raw microarray image data were processed with the Image Analysis / Feature Extraction software G2567AA (Version A.7.5, Agilent technologies). Data analysis was carried out on the Rosetta Inpharmatics platform Resolver

(Rosetta Biosoftware, Built 5.1). A color-swap dye reversal experimental setting was applied [36]. Ratio profiles comprising single hybridizations were combined in an error-weighted fashion to create ratio experiments. A two fold change expression cut-off ratio was applied together with anti-correlation of ratio profiles rendering the microarray analysis set highly significant (P-value > 0.01), robust and reproducible.

### Bioinformatics

A *S*. Typhimurium specific weight matrix which predicts the affinity of phosphorylated CpxR for a given DNA sequence was developed using as input files the promoter regions of operons *cpxRA*, *cpxP*, *motABcheAW*, *tsr*, *spy*, *yihE-dsbA*, *degP* (*htrA*), *ppiA*, *yccA* and *rpoE-rseABC*. These promoters were chosen because among the best defined CpxR targets [37] these were identified to be strongest affected in SHS01 under invasion-inducing conditions by our microarray analysis (S1 Table). We aligned 500-bp of the promoter regions upstream of the start codons of these operons with the motif-finding program Target Explorer which is based on the Gibbs sampling algorithm [38]. A conserved 15-bp motif was identified for each promoter (see S2 Table). These 10 motifs were used to calculate a *S*. Typhimurium specific weight matrix which was then used to search both strands of the genome (GenBank accession number AE006468) and the pSLT plasmid (GenBank accession number AE006471) of *S*. Typhimurium LT2 with the programs ScanACE [39], Prodoric Virtual Footprint [40, 41] and GeneSoap [42].

### RNA extraction and RT-qPCR

Total RNA from cells grown under invasion inducing conditions was isolated after stabilizing the RNA with RNAprotect (Qiagen, Hilden, Germany). Cell pellets were resuspended in 10 mM Tris, 1 mM EDTA containing 2 mg ml-1 lysozyme and incubated at 37°C for 30 min. Cell lysis solution (Qiagen, Hilden, Germany) was added and the mixture was incubated at 65°C for 5 min and at room temperature for 10 min. After the addition of precipitation solution (Qiagen, Hilden, Germany) and incubation on ice for 5 min cell debris, proteins and DNA were pelleted. The RNA containing supernatant was mixed with ethanol and loaded on a spin column (Promega, WI, USA). Further RNA purification and DNase digestion was done as described by the manufacturer (SV total RNA isolation system, Promega, WI, USA).

In total, 1 mg of total RNA was reverse transcribed with the reverse transcription kit (Qiagen, Hilden, Germany). Diluted cDNA samples were used as templates in Real-time qPCR analysis using specific primer pairs and SYBR Green fluorescent dye. Real-time PCR was performed using PowerSYBR Green PCR Mastermix on a 7500 Fast PCR Cycler (AppliedBiosystems, Carlsbad, CA, USA). Uniformity of the product was checked for every PCR by the determination of a dissociation curve. Pairs of primers with lengths of 19–21 nucleotides were optimized for use at an annealing temperature of 51°C. Each primer pair amplified a fragment of 150–250 bp. Relative expression ratios were determined by the DDCt method as described [43] and normalized to the level of 16S-RNA as a constitutive control. Experiments were repeated nine times with biological triplicate performed in technical triplicate reactions/cDNA dilution.

### Expression and purification of the CpxR protein

*Salmonella* His6-CpxR was essentially overproduced and purified as described for the *E*. *coli* protein from strain BL21DE3<pLysS|pIStmCpxR> using Ni-NTA agarose (Qiagen) and Protino Ni-TED 2000 column kit (Macherey-Nagel) [6].

### Gel shift assay

The promoter regions of the genes *eco* (301 bp), *pgtE* (251 bp), *pocR* (301 bp), *ssrB* (301 bp) and *tatA* (251 bp) were generated by PCR using the *Salmonella* SL1344 genomic DNA as template. As control a 156 bp fragment of the *Salmonella* cpxP promoter region without the CpxR~P recognition motif was used. Binding reactions were performed as previously described for RovM using the purified His6-CpxR [44].

In brief, phosphorylation of His6-CpxR was carried out using 50 mM acetyl phosphate in a phosphorylation buffer (100 mM Tris, pH 7.4, 10 mM MgCl2, 125 mM KCl) for 1 h at 30°C. DNA fragments (0.15 pmol) were mixed with phosphorylated His6-CpxR (0–23 pmol) in a 10 μl reaction mixture containing DNA binding buffer (10 mM Tris, pH 7.4, 10 mM MgCl2, 100 mM KCl, 10% glycerol, 2 mM dithiothreitol, 30 μM BSA). The binding reaction was carried out for 20 minutes at room temperature and subsequently loaded on a 4% non-denaturing acrylamide gel and stained with ethidium bromide.

### Motility

SL1344, NOS01, SL1344<pSSS11> and NOS01<pSSS11> were grown overnight in TB pH 7.0 and then freshly diluted 1 : 50 into TB pH 7.0. The cultures were grown until OD600 = 1.0 and then diluted to an OD600 of 0.1. 1 μl of the cultures were semi-stabbed into tryptone motility agar plates (0.3% bacto agar) of pH 8.0. The plates were incubated at 30°C for 10 hours. Strain KT04 (SL1344 *fliC*, *fliB*) was used as negative control.

### Polymyxin B survival assay

The sensitivity to polymyxin B was performed as described previously [45]. The overnight cultures of SL1344, NOS01 and NOS01 <pIStmCpxR> were diluted into fresh LB pH 8.0 medium with the colony-forming units (CFU) being kept constant at 4 x 105 CFU ml-1. Cells were allowed to grow to OD600 = 0.14 at 37oC. 1 ml of the cultures were incubated with six different concentrations (ng ml^-1^) of polymyxin B: 0, 100, 101, 102, 103, 104, 105, 106 in polypropylene tubes and allowed to stand at 37°C for 1 h. After incubation cultures were washed twice with 1 x PBS to remove the remnant polymyxin B hindering the growth of bacteria when plated. During plating, 20 μl of the bacterial cells treated with the appropriate concentrations of polymyxin B was mixed with 20 ml of molten Tryptic Soya Agar (55°C) and the plates were incubated overnight at 37°C. All experiments were done in triplicates. For control purpose the experiments were performed for a *phoP*::*kan* strain. The number of CFU from each plate was counted and the % survival was determined taking the CFU counted from the plates with non-polymyxin B treated bacteria.

### DNaseI footprinting

For DNase I footprinting, different segments of the tatA and ssrB promoter regions were amplified by PCR using a digoxigenin (DIG)-labelled primer and a non-labelled primer. Different primer combinations were chosen for the footprinting analysis of coding and non-coding strands (Table 2). The amplified promoter regions were 529 and 530 bp for *ssrB*-A (DIG-coding strand) and *ssrB*-B (DIG-Non-coding strand) respectively. The amplified promoter region was 524 bp, for *tatA*-A (coding strand) and *tatA*-B (non-coding strand). PCR fragments were purified by Nucleospin plasmid kit (Macherey-Nagel) and incubated with the purified phosphorylated His6-CpxR protein in 20 μl of DNA-binding buffer as described for the gel shift assays. The PCR products were digested with DNaseI of an appropriate dilution and the resulting products were separated and visualized as described [44]. The protected bands were identified by comparison with a sequence ladder generated with the same DIG-labelled primer used for PCR amplification of the fragment by using the Thermo Sequenase cycle sequencing kit (USB).

**Table 2:** Oligonucleotides used in this study.

**Primer Name**	**Sequence (5’-3’)**	**Construct / Purpose**
cpxA5-STM-PS1	ATTGCGTGGTCGCGGCTATCTGATGGTTTCCGCTTCATGAGTGTGTAGGCT	SHS01
cpxA3-STM-PS4	CGAGATAAAAAATCGGCCTGCATTCGCAGGCCGATGGTTTATTCCGGGGAT	NOS01
cpxR5-STM-PS1	GACGCCTGATGACGTAATTTCTGCCTCGGAGGTACGTAAACAGTGTGTAGGCTGGAGCTGCTCC	NOS01
cpxR3-STM-PS2	CAACAAGAAGATGGCGAAGATGCGCGCGGTTAAACTTCCTACATATGAATATCCTCCTTAG	VSM01
cpxAR5-STM-PS1	GACGCCTGATGACGTAATTTCTGCCTCGGAGGTACGTAAACAATGTGTAGGCTGGAGCTGCTTC-	VSM01
cpxAR3-STM-PS2	ATCGGCCTGCATTCGCAGGCCGATGGTTTTTAGGTTCGCTTGTACATATGAATATCCTCCTTAG	pIStmCpxR
NdeI_STMcpxR	ATCATATGAATAAAATCCTG	pSSS11
BamHI_SCpxRA	CGGCGCGGATCCATGAATAAAATCCTGTTAGTTGATGA	pSSS11
SCpxRA_SalI	GGGGGCGTCGACTTAGGTTCGCTTGTACAGCGGTAGCC	EMSA
P*cpxR-*Fw	GCCGTCAAACATATGATT	EMSA
P*cpxR*-Rev	TCATTGTTTACGTACCTCCG	EMSA
P*eco*-Fw	CCGATAGAGGTAAATGCTG	EMSA
P*eco*-Rev	TTCATTTGATTGTTCACAGTAT	EMSA
p*pgtE*-Fw	GACAACATCAGCAACGATG	EMSA
p*pgtE*-Rev	CATTTCTCTTGTCCTCATATTC	EMSA
p*pocR*-Fw	CCTGTTATCGGCGCCTGTGCCGGAGCAGCCATATATC	EMSA
p*pocR*-Rev	CATGATAAAACCCCTCAGTTAATTTATTGTTATAAAC	EMSA
P*ssrB*-Fw	GATATGGTCATTAATAGCAAG	EMSA
P*ssrB*-Rev	ATTTTGCTGCCCTCGCGA	EMSA
P*tatA*-Fw	CAACCGCCCTGAATGGG	EMSA
P*tatA*-Rev	ACATGTTCCTCTGTGATAGA	EMSA
P*cpxP*-control-fw	ATGAATAAAATCCTGTTAGTTGA	EMSA
P*cpxP*-control-rev	AAAAGTAAATCGATGCTGTCAT	EMSA
*ssrB*-A-Fw	DIG-CCTCTTGCTGGCTGATATT	footprinting
*ssrB*-A-Rev	Non-DIG-ATATACTCTTGTTGGTATGCT	footprinting
*ssrB*-B-Fw	Non-DIG-CATGTTTGTGGCACTATCC	footprinting
*ssrB*-B-Rev	DIG-CGTCTACTAATAAGATCTTATA	footprinting
*tatA*-A-Fw	DIG- AGTTGTCTGGCTGGTTGG	footprinting
*tatA*-A-Rev	Non-DIG-TTCGCTAAAACCGATATCAAA	footprinting
*tatA*-B-Fw	Non-DIG-GCGCCGTTCTGGGTCG	footprinting
*tatA*-B-Rev	DIG-CAACAATCAACAACTGCCAA	footprinting

### Cell growth on 1,2-propandiol

Anaerobic growth on 1,2-propaendiol was determined according to an established protocol [24]. In brief, tubes were filled with NCE glycerol with or without 80 mM propanediol and preincubated in an anaerobic chamber (Oxoid) with N2 gas for 24 h. Cells were grown aerobically in NCE glycerol to stationary phase, washed in NCE and diluted to an turbidity at 650 nm of 0.1. The tubes were crimp capped and flushed with N2 gas. The cultures were incubated at 37°C with shaking and turbidity was monitored with a tube spectrophotometer (Riele PM310) at 650 nm.

### Statistical analyses

Statistical analyses were performed using the Student’s t-test (two-tailed). A P-value < 0.05 was considered significant.

## Results

### CpxA-mediated dephosphorylation of CpxR is required for *hilA* expression

**Fig 1:**
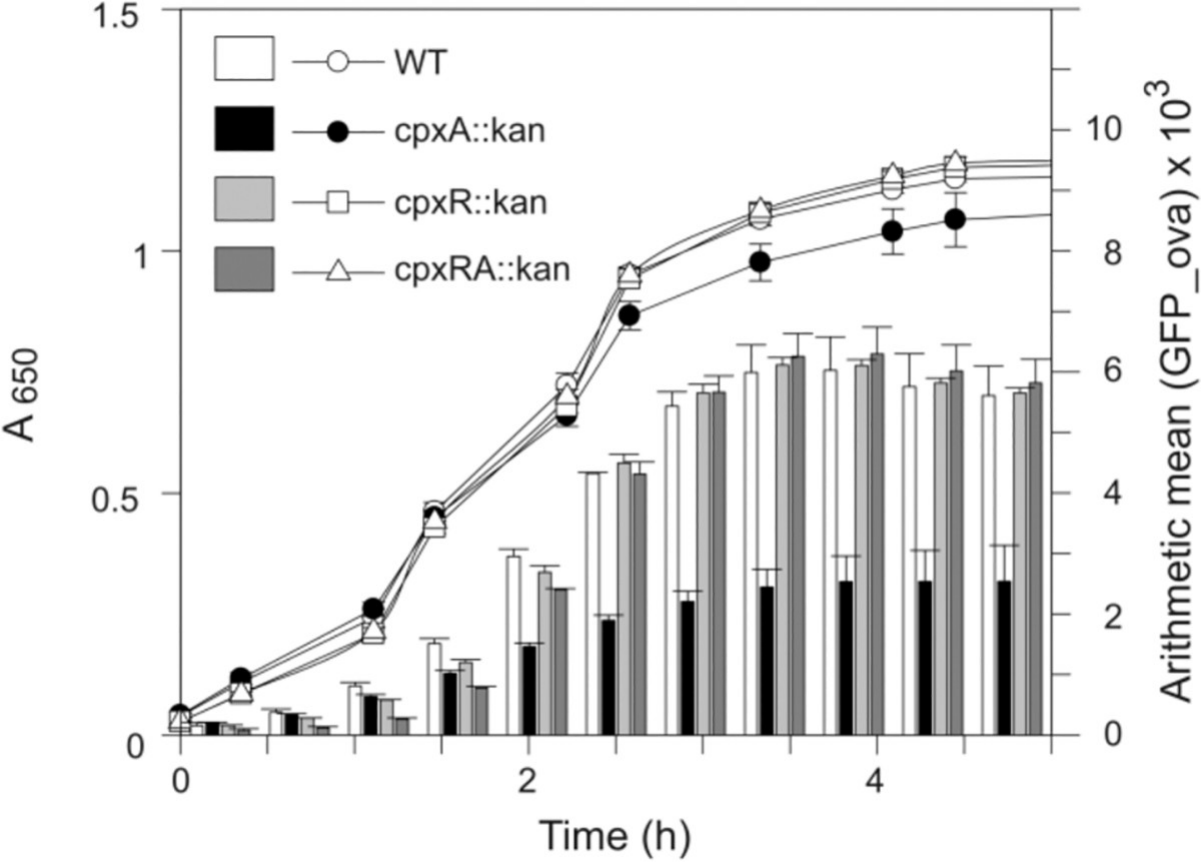
CpxR activity is responsible for *hilA* transcription. Fluorescence was determined for the SL1344 wild type (white symbols), *cpxA* (black symbols), *cpxR* (silver symbols) or *cpxRA* (dark gray symbols) strains transformed with a plasmid carrying a GFP fusion to the hilA promoter (pD.2E) [52]. Shown are the growth curves (circles) and expression results (bares) for cultures grown under invasion inducing conditions. Shown are the means ± S.E.M. of biological triplicates (t-test).

The Cpx envelope stress system is implicated in the invasion process of *Salmonella* into non-phagocytic cells which depends on the SPI-1 T3SS with HilA as the key transcriptional regulator [19, 20, 46]. The expression of *hilA* at low pH requires the sensor kinase CpxA but not the response regulator CpxR [47]. We asked whether *hilA* expression might also be dependent on the Cpx envelope stress system under *in vitro* conditions that mimic invasion. Therefore, we investigated the expression of a plasmid-coded hilA-GFP fusion under invasion inducing conditions in a high-salt LB medium under low oxygen tension for *S*. Typhimurium knock out strains SHS01 (*cpxA*::*kan*), NOS01 (*cpxR*::*kan*) and VSM01 (*cpxRA*::*kan*) in comparison to the wild-type strain SL1344. We found under these invasion inducing conditions the expression of *hilA* to be dependent on CpxA but not on CpxR (Fig 1) and confirmed that this effect is not traceable in standard LB medium (S1 Fig) [20]. Interestingly, we observed no effect on *hilA* expression for the *S*. Typhimurium *cpxAR* double deletion mutant VSM01 (Fig 1, dark grey bars). It is well established that CpxR can be phosphorylated independent on CpxA by the small phosphodonor acetyl-phosphate *in vivo* [48–50] and that CpxA acts under non-inducing conditions as a phosphatase of phosphorylated CpxR [6, 51]. We would like to propose that CpxR is constitutively phosphorylated in a *S*. Typhimurium *cpxA* knock out grown under invasion inducing conditions leading to repression of *hilA* transcription. Therefore, our finding suggests that dephosphorylation of CpxR by CpxA might be critical for *hilA* expression.

### Genome wide screen for Cpx interactions

**Fig 2:**
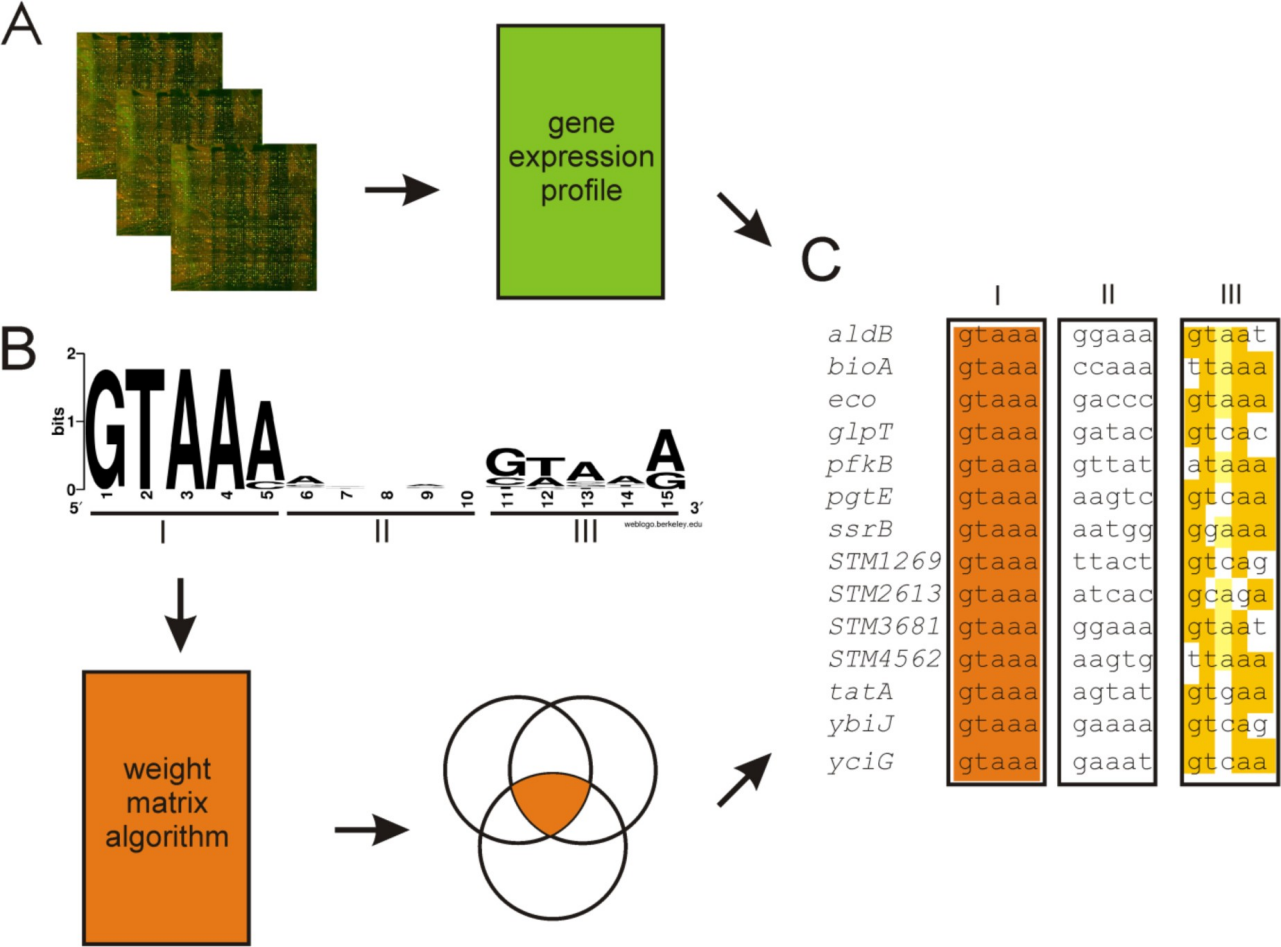
Genome wide identification of novel CpxR dependent genes in *S*. Typhimurium. A. Global transcriptional analysis was performed by comparing the gene expression profile of a *cpxA* mutant (SHS01) to a wild-type strain (SL1344) grown under invasion inducing conditions. Genes for which the ratio of the *cpxA*-null strain to the wild-type strain is e^4^ fold with a P value of <0.005 were clustered using Tibco Spotfire DecisionSite default. B. Bioinformatics-based screening for direct CpxR targets. The sequence logo for the CpxR~P recognition weight matrix in *S*. Typhimurium is given with the left (I) and right pentamers [28] and the 5-bp spacer marked by roman numerals. The base conservation measured in bits is shown as the relative height of each base [54]. C. Potential novel Cpx regulon members of *S*. Typhimurium identified by global transcriptional analysis and bioinformatic screening. The left (I) and right [28] pentamers and the 5-bp spacer [28] are displayed as a heat-map to show bases of high conservation (orange) from degenerate regions (light grey/white). The genes controlled by these promoters are indicated to the left of the sequences.

From our above results we conclude that CpxR is constitutively active in a *cpxA* knock out strain (SHS01) under invasion inducing conditions. Consequently, we determined the extent of the Cpx envelope stress system in *S*. Typhimurium by global transcriptional analysis of a *cpxA* knock out strain (SHS01) grown under invasion inducing conditions with high osmolarity (0.3 M NaCl) and found 392 genes to be differently regulated (S1 Table). The CpxR target gene *cpxP* was the most strongly affected gene supporting our hypothesis that deletion of CpxA results in high level of phosphorylated CpxR. This supports the widely accepted hypothesis that CpxP can be only switched on by activation via the stress pathway [53]. To predict among these genes those that are under direct control of CpxR, we compared the data of the global transcriptional analysis with a bioinformatic approach using a *S*. Typhimurium specific CpxR recognition motif (Fig 2B; S2 Table). This *S*. Typhimurium specific CpxR recognition motif was generated by using among the best defined CpxR targets in *E*. *coli* [37] the promoter regions of those 10 CpxR targets that were strongest affected in the *S*. Typhimurium *cpxA* strain (SHS01) under invasion inducing conditions as determined by the transcriptional analysis (S1 Table) (*cpxRA*, *cpxP*, *motABcheAW*, *tsr*, *spy*, *yihE-dsbA*, *degP*, *ppiA*, *yccA* and *rpoE-rseABC*). For each promoter a conserved 15-bp motif was identified (S2 Table) and these 10 motifs were used to calculate a *S*. Typhimurium specific weight matrix (Fig 2B).

We employed this weight matrix to screen the whole *S*. Typhimurium LT2 genomic and pSLT sequences for possible CpxR recognition motifs by the use of three different motif-finding programs applying different algorithms each: ScanACE, Prodoric Virtual Footprint and GeneSoap [39, 40, 42]. 106 potential CpxR recognition motifs were commonly identified by all three programs in an appropriate distance of less than 500 bp to an annotated open reading frame (S3 Table). The comparison of bioinformatics and global transcriptional analysis data resulted in 25 possible CpxR target genes (Fig 2C). Along with 11 known genes (*chaA*, *cpxP*, *cpxR*, *dsbA*, *motA*, *psd*, *tsr*, *ppiA*, *rpoE*, *spy*, *yccA*) [37, 55–57] 14 potential novel CpxR target genes could be identified (*aldB*, *bioA*, *eco*, *glpT*, *pfkB*, *pgtE*, *ssrB*, *STM1269*, *STM2613*, *STM3681*,*STM4562*, *tatA*, *ybiJ*, *yciG*) (Fig 2C). These potential novel CpxR target genes were clustered into genes with uncharacterized products (*STM1269*, *STM2613*, *STM3681*, *STM4562*, *ybiJ*, *yciG*), products implicated in metabolism (*aldB*, *bioA*, *glpT*, *pfkB*) or Salmonella virulence (*eco*, *pgtE*, *ssrB*, *tatA*). For our further studies we focused on four genes that have been demonstrated to be important for *Salmonella* virulence: *eco*, *pgtE*, *ssrB* and *tatA*.

The *eco* gene encodes the serine protease inhibitor ecotin. Ecotin orthologues are present in many Gram-negative bacteria and have been shown to be important for protecting the bacteria against eukarytotic proteases that have translocated across the damaged outer membrane [58]. PgtE is a member of the omptin family of outer membrane aspartic proteases identified in Gram-negative bacteria [23]. PgtE and its closest homologue of Pla of Yersinia pestis attack on the innate immunity for instance by means of inactivating antimicrobial peptides or by affecting the plasminogen/plasmin system by cleavage of the plasminogen activator inhibitor 1 [59–61]. SsrB is the response regulator of the SsrAB two-component system which is essential for the coordinated expression of a second T3SS encoded on the SPI-2 and almost all of its accessory effector proteins [62, 63]. Salmonella requires the SPI-2 T3SS for intracellular survival and persistence in macrophages [62, 64]. The tatA gene belongs to the operon for the twin-arginine translocation [23] complex TatABC which promotes the secretion of folded proteins across the cytoplasmic membrane and has recently been demonstrated to be implicated into Salmonella invasion and resistance to antimicrobial peptides [22, 65].

**Fig 3:**
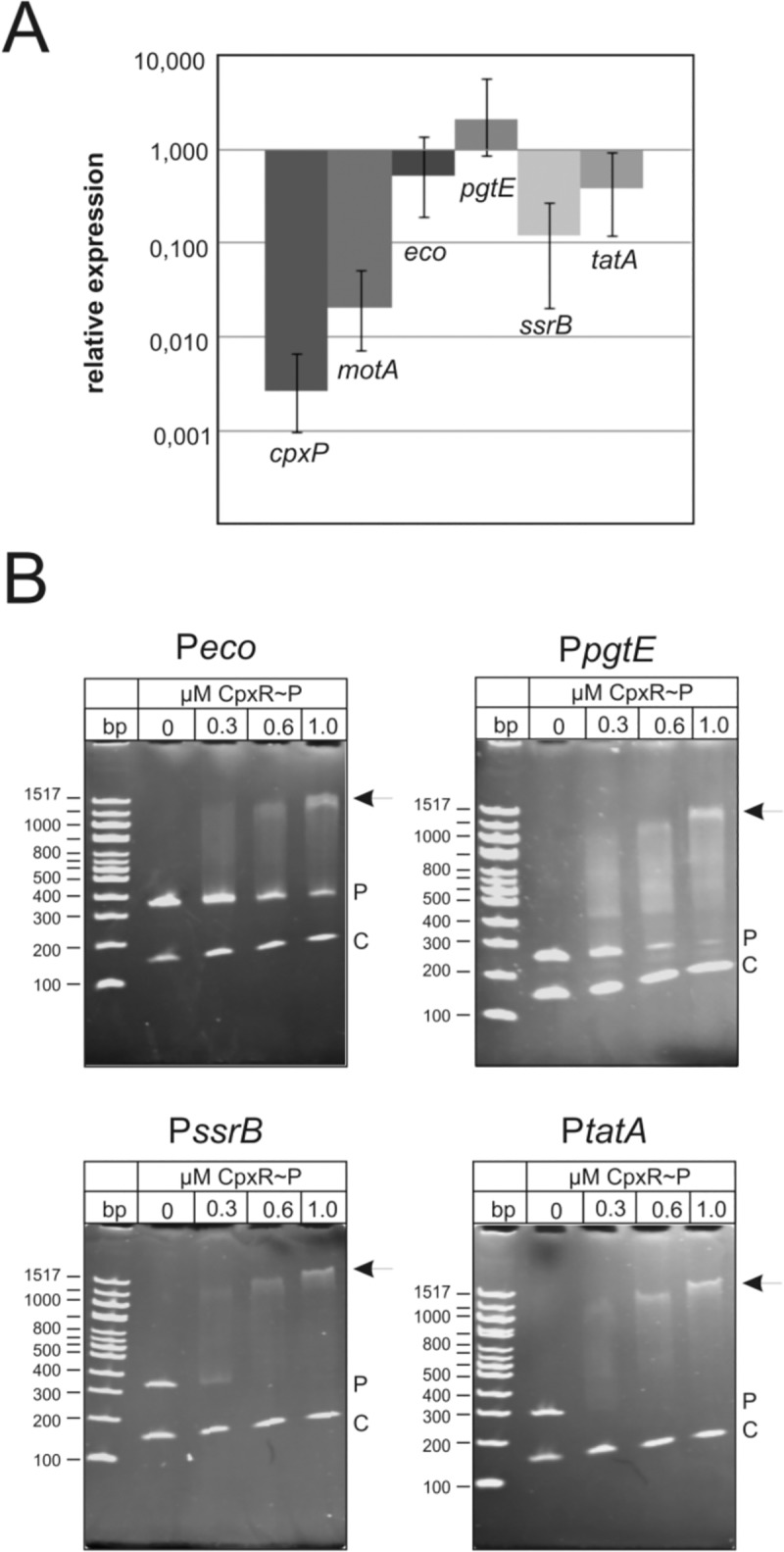
The Salmonella virulence determinants *eco*, *pgtE*, *ssrB* and *tatA* are under direct CpxR control. A. qRT-PCR analysis of CpxR target genes in the S. Typhimurium *cpxR* mutant NOS01. RNA samples were prepared from bacteria grown under invasion inducing conditions. In addition to the relative expression levels of *cpxP* and *motA*, the levels of *eco*, *pgtE*, *ssrB* and *tatA* were determined in NOS01 (sample) in comparison with the parental strain SL1344 (control) according to the formula ratio = 2^-[ΔCt, sample– ΔCt, control]^ [43]. Columns indicate the mean values of one representative of biological triplicates together with the RQmin and RQmax. **B.** EMSA analysis verifies CpxR binding to the *eco*, *pgtE*, *ssrB* and *tatA* promoters. Indicated promoter fragments (p) were incubated without or with increasing amounts of the purified and phosphorylated CpxR protein. The DNA-CpxR~P complexes were separated on 4% polyacrylamide gels. The corresponding molecular weights are indicated on the left. The positions of promoter fragments are indicated (p), arrows show the higher molecular weight DNA-CpxR~P complexes. A fragment of *cpxP* promoter region without the CpxR~P binding motif (-151 to -297) was used as negative control (c).

To validate the identification of *eco*, *pgtE*, *ssrB* and *tatA* as direct CpxR target genes in S. Typhimurium, real-time qPCR and electrophoretic mobility shift assays (EMSA) were performed (Fig 3). Cells were grown under invasion inducing conditions and harvested at the end of mid log phase for RNA preparation. The genes *cpxP* and *motA* served as controls. The expression of all tested genes was dependent on CpxR (Fig 3A). We found *eco* (1.9 fold), *ssrB* (8.2 fold) and *tatA* (2.6 fold) to be under positive and pgtE (-2.1-fold) to be under negative CpxR control. In order to demonstrate direct binding of CpxR to the promoter regions of *eco*, *pgtE*, *ssrB* and *tatA* we performed electrophoretic mobility shift assays (EMSA) with purified, phosphorylated His6-CpxR (CpxR~P) tagged protein (Fig 3B). The mobility of all DNA fragments covering the single promoter regions were retarded in the presence of phosphorylated His6-CpxR (Fig 3B). To confirm the species specificity displayed by CpxR~P in binding to *tatA* promoter of *S*. Typhimurium, we performed the EMSA for *E*. *coli tatA* promoter using CpxR~P and found no significant shift (S2 Fig). Together, these results demonstrate that the *eco*, *pgtE*, *ssrB* and *tatABC* operons are direct CpxR targets in *S*. Typhimurium.

### The Cpx envelope stress system activates *Salmonella* motility

**Fig 4:**
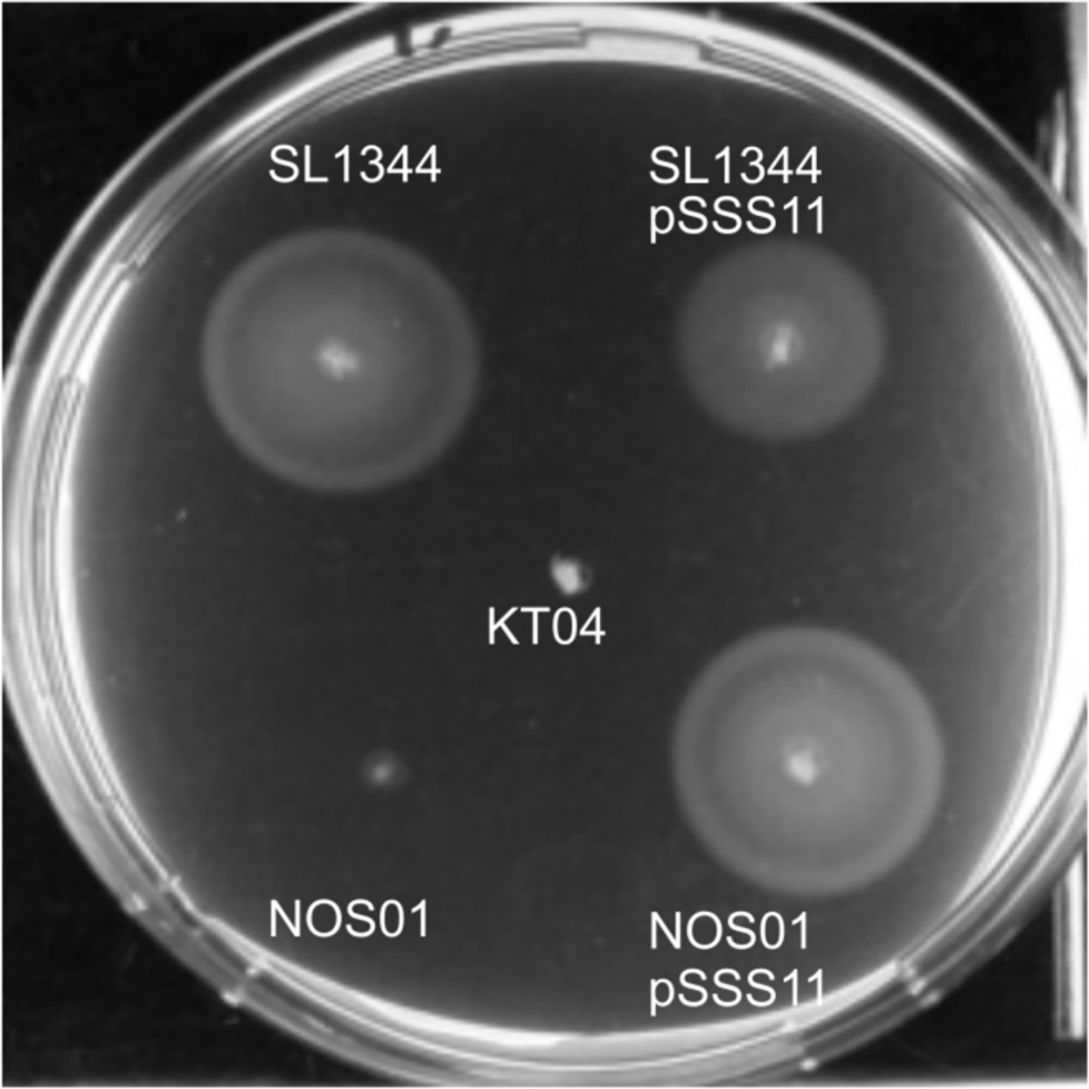
*S*. typhimurium is immotile in the absence of CpxR. An equal number of freshly grown *S*. Typhimurium wild type SL1344, the *cpxR* mutant NOS01, and the *cpxRA* overexpressing strains SL1344<pSSS11> and NOS01<pSSS11> was spotted onto tryptone swarm soft agar plates pH 8.0 at 30°C for 10h. KT04 (SL1344 *fliC*, *fljB*) was used as negative control.

To our surprise, global transcriptional analysis and qRT-PCR revealed *motA* to be under positive control (50 fold) of the Cpx envelope stress system. In contrast, the genes for the flagellar motor (*motABcheAW*) are under negative CpxR control in *E*. *coli* [37, 66]. We verified phenotypic difference between both closely related organisms by motility assay (Fig 4). Motility of *S*. Typhimurium was drastically repressed in a *cpxR* deletion strain and could be restored by the overexpression of CpxR. This finding underscores the remarkable difference in core gene regulation between *Salmonella* and *Escherichia* as has also been described for the *tolQRA* cluster, SlyA, DegP, RpoS stability and copper homeostasis [67–71].

### A *cpxR* mutant displays increased sensitivity to polymyxin B

**Fig 5:**
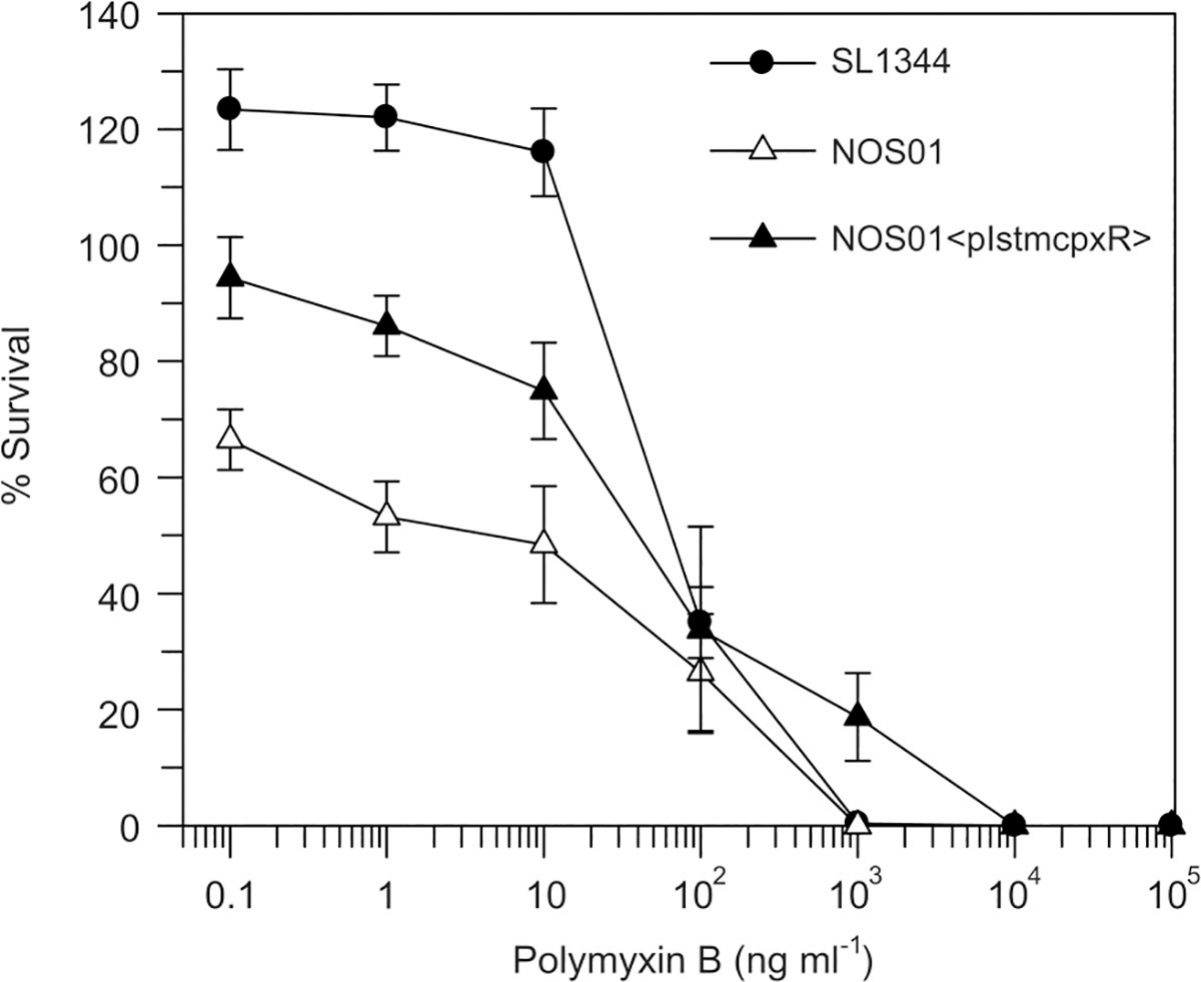
A *cpxR* mutant displays increased sensitivity to polymyxin B. *S*. Typhimurium wild type SL1344 (black circles), the cpxR mutant NOS01 (white triangles), and the complementation strain NOS01<pIStmCpxR> (black triangles) were treated with indicated polymyxin B concentrations at 37°C for 1 h. Cells were centrifuged and washed twice with 1x PBS before plating to determine their viability. The % survival confers to the non-polymyxin B treated bacteria at time zero.

We identified the omptin PgtE as a direct Cpx target. PgtE confers resistance towards the cationic antimicrobial peptides (CAMPs) protamine and polymyxin B [60]. Very recently, it has been proven that the Cpx-system contributes to resistance to the CAMPs protamine, magainin-2 and melittin through regulated expression of the two Tat-system dependent amidases AmiA and AmiC [22]. AmiA and AmiC do not contribute to bacterial resistance to the CAMPs HNP-1 and polymyxin B [22]. In order to investigate whether the Cpx pathway also contributes resistance to polymyxin B, we performed a polymyxin B resistance assay. As shown in Fig 5 resistance against polymyxin B is reduced in a *cpxR* deletion strain and could be partially restored by the overexpression of CpxR indicating that the Cpx-system modulates the resistance to CAMPs in *S*. Typhimurium by regulating PgtE.

### Detection of the CpxR binding sites for the *ssrB* and *tatABC* promoter regions

**Fig 6:**
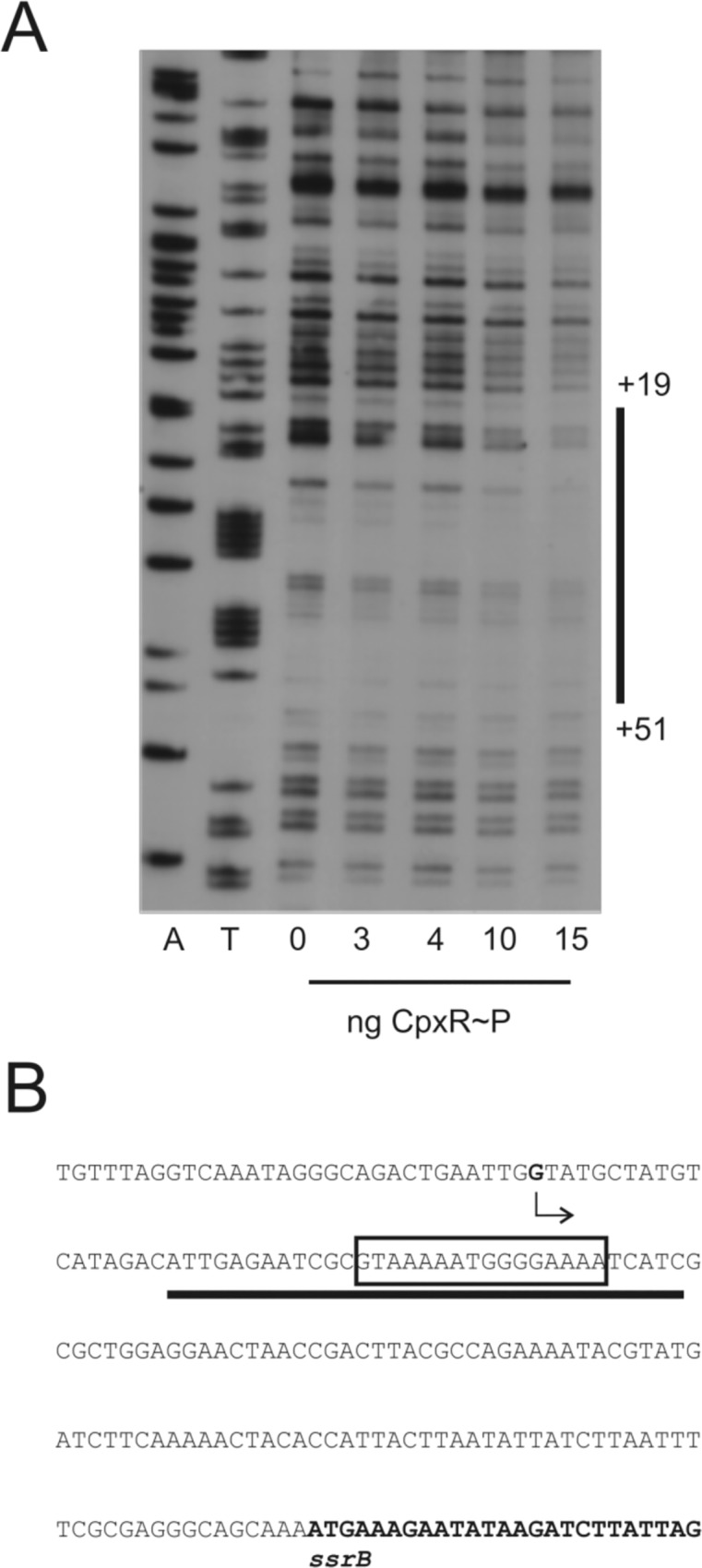
Determination of CpxR binding sites for the *ssrB* promoter region. A. DNase I footprinting analysis of the *ssrB* promoter performed with the probe for the non-coding strand and increasing amounts of 6His-CpxR~P protein (see Experimental Procedures). The CpxR~P binding site is indicated by a solid line. Numbers are relative to the transcription start site of the *ssrB* gene obtained by [73]. B. DNA sequence of the *ssrB* promoter region. The sequence corresponds to the coding strand of the fragment used for DNase I footprinting analysis presented in (A). Boxed bases indicate the CpxR~P recognition motif. The reported transcription start site [73] is indicated by an arrow and the gene coding region in bold. The black line indicates the identified CpxR~P binding site.

To map the precise location of the CpxR binding sites for the *ssrB* and *tatABC* promoter regions we performed DNase I footprint assays. For the *ssrB* promoter, a 250 bp DIG-labelled DNA fragment including the CpxR recognition motif was incubated with increasing concentrations of purified and phosphorylated His6-CpxR (CpxR~P) prior to DNaseI on the non-coding strand from positions +19 to +51 downstream of the transcriptional start site treatment (Fig 6A). The analysis of both strands revealed one binding region for phosphorylated His6-CpxR overlapping the CpxR recognition motif (Fig 6A and 6B). We checked the coding strand of *ssrB* for binding of CpxR~P but did not observe significant binding (S3 Fig). This result also indicates that phosphorylated His6-CpxR binds to its consensus motif on *ssrB*’s non-coding strand specifically and serves as a direct regulator of *ssrB*’s expression.

**Fig 7:**
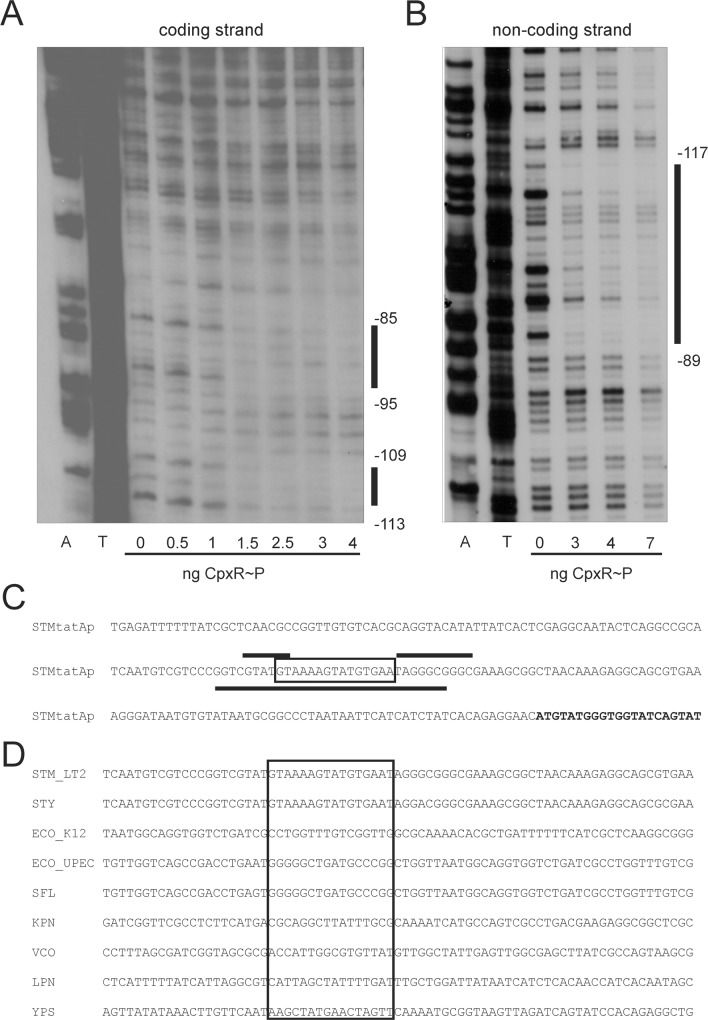
Determination of CpxR binding sites for the *tatABC* promoter region. DNase I footprinting analysis of the *tatA* promoter performed with probes for the coding strand (A) and non-coding-strand (B) with increasing amounts of 6His-CpxR~P protein (see Experimental Procedures). Solid vertical lines correspond to regions protected by the 6His-CpxR~P protein. Numbers are relative to the translational start site of the *S*. Typhimurium *tatA* gene. C. Comparison of the promoter sequence of the *tatABC* operon of *S*. Typhimurium and *E*. *coli* (ECO). Boxed bases indicate those nucleotides that were identified by weight matrix anlaysis as a CpxR~P recognition motif. The reported transcription start site for the *E*. *coli* promoter [72] is indicated by an arrow. The gene coding sequences are highlighted in bold letters. Bases that are protected by CpxR~P are indicated by black lines. Lines on top of the bases show protection regions identified for the coding strand and lines below the bases show regions identified for the non-coding strand.

To investigate the binding of CpxR to the *tatABC* promoter region, a 250 bp DIG-labelled DNA fragment including the CpxR recognition motif was subjected to DNase I footprint analysis (Fig 7A and 7B). In contrast to the *ssrB* promoter region, binding regions for phosphorylated His6-CpxR were located on both strands (Fig 7A and 7B). The binding regions on the coding strand flanked the Cpx recognition motif (Fig 7A and 7C) and overlapped the CpxR recognition motif on the non-coding strand (Fig 7B and 7C).

Our data clearly assign the *tatABC* operon as a direct Cpx target in *S*. Typhimurium. In contrast, the *tatABC* operon of *E*. *coli* has been described to be constitutively expressed [72]. In order to clarify the discrepancy of *tatABC* regulation between *S*. Typhimurium and *E*. *coli* we compared the structure and promoter sequences of the *tatABC* operons of both organisms. The *tatABC* promoter of *E*. *coli* is substantially shorter than that of *S*. Typhimurium and lacks the Cpx recognition motif (Fig 7C). We asked whether the *tatABC* operon might be CpxR-dependent regulated in pathogenic *E*. *coli* strains or other pathogenic genera like *Shigella*, *Yersinia* or *Legionella*. The Cpx recognition motif could only be identified in the *tatABC* promoter region of different Salmonella strains (Fig 7D) indicating an adaptation of Salmonella to a specific niche that depends on a substrate of the Tat complex. D. Comparison of the promoter sequence of the *tatABDC* operon of *S*. Typhimurium (STM), *S*. Typhi (STY), *E*. *coli* MG1655 (ECO_K12), *E*. *coli* UTI89 (ECO_UPEC), *Shigella flexneri* (SFL), *Klebsiella pneumophila* (KPN), *Vibrio cholerae* El Tor (VCO), *Legionella pneumophila lens* (LPN) and *Yersinia pseudotuberculosis* (YPS). Given are the homologue *tatABCD* promoter regions of each strain. Boxed bases indicate those nucleotides that were identified by weight matrix anlaysis as a CpxR recognition motif for *S*. Typhimurium.

### The Cpx envelope stress system inhibits anaerobic growth on 1,2-propanediol

From the above results we hypothesized, that the efficient transport of one or several specific substrates of the Tat-system might be the reason that the *tatABC* operon is adirect CpxR target in *Salmonella* but not in other Gram-negative pathogens. The α-subunit of the tetrathionate reductase TtrA was described as a *Salmonella* specific Tat-dependent substrate [74, 75]. The intriguing *ttrBCA* operon also includes TtrB subunit with a Tat motif where the invariant arginines are swapped with lysines [76]. The tetrathionate reductase is a membrane-bound enzyme that contains the guanine dinucleotide cofactor as a prosthetic group and confers anaerobic respiration of *Salmonella* on tetrathionate as terminal electron acceptor [24, 74, 77, 78].

**Fig 8:**
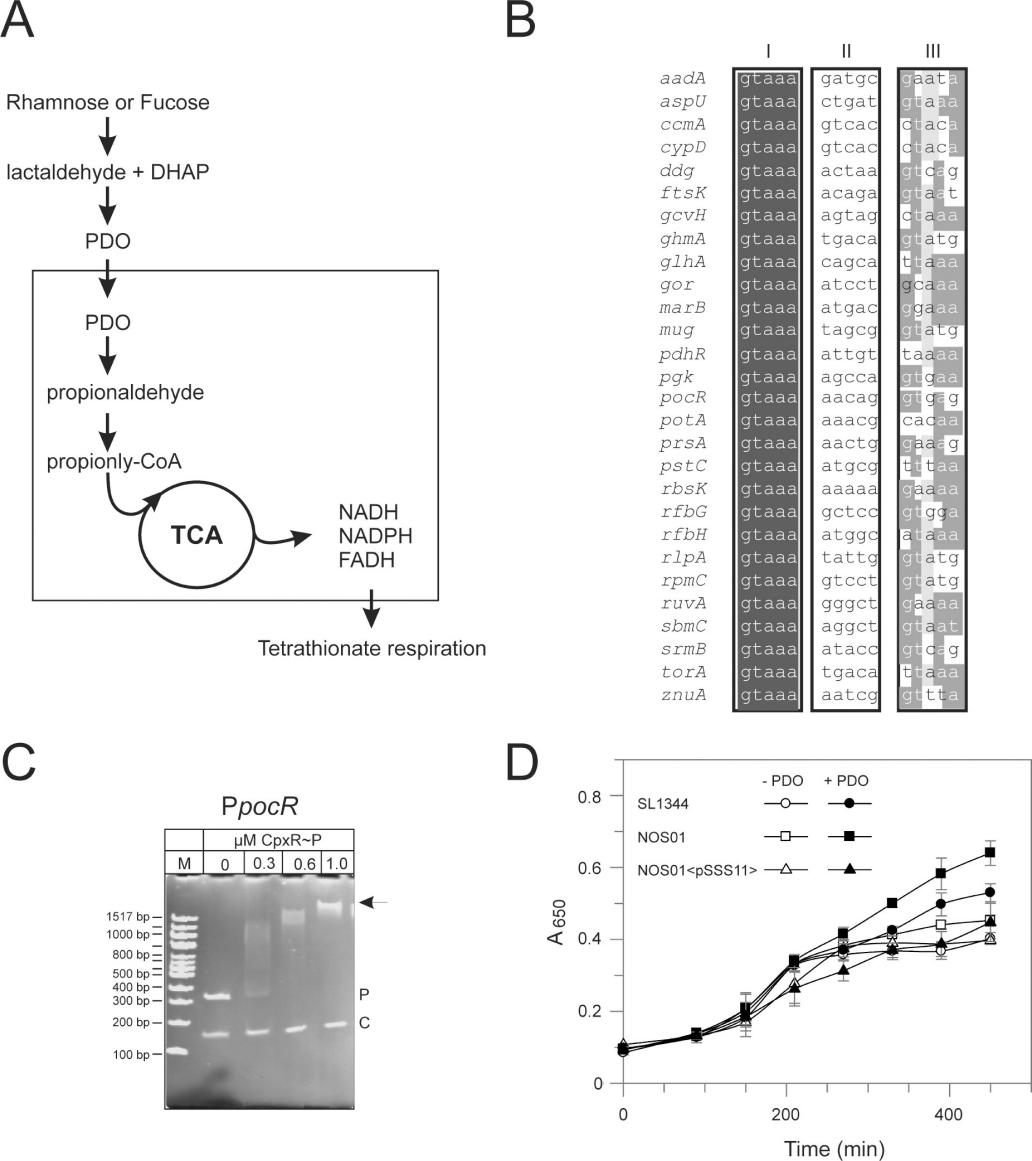
Cpx envelope stress system enhances 1,2-propanediol-dependent anaerobic growth of *S*. Typhimurium. A. The diagram outlines the principle steps in the metabolism of 1,2‐propanediol (PDO). B. Potential novel Cpx regulon members of *S*. Typhimurium identified by bioinformatics based screening. The left (I) and right [28] pentamers and the 5-bp spacer [28] are displayed as a heat-map to show bases of high conservation (dark grey) from degenerate regions (light grey/white) (compare Fig 2). The genes controlled by these promoters are indicated to the left of the sequences. C. EMSA analysis verifies CpxR binding to the *pocR* promoter. PocR promoter fragment (p) was incubated without or with increasing amounts of the purified and phosphorylated CpxR protein. The DNA-CpxR~P complexes were separated on 4% polyacrylamide gels. The corresponding molecular weights are indicated on the left. The positions of the promoter fragments are indicated (p), arrows show the higher molecular weight DNA-CpxR~P complexes. A fragment of the *cpxP* promoter region without the CpxR~P binding motif (-151 to -297) was used as negative control (c). D. Stimulation of anaerobic growth by 1,2-propanediol (PDO) was determined according to an established protocol [24]. Cells of wild-type *S*. Typhimurium strain SL1344, the *cpxR* mutant NOS01, and the *cpxRA* overexpressing strain NOS01<pSSS11> were grown anaerobically on minimal NCE medium supplemented with 0.2% yeast extract as carbon source with or without 80 mM PDO as energy source. Shown are the means ± S.E.M. of biological quadruples (t-test).

Tetrathionate respiration is coupled with the degradation of 1,2-propanediol and ethanoleamine, respectively, that act as electron donors and that are both abundant in the human intestine [24, 76]. Interestingly, 1,2-propanediol was described to repress *hilA* expression [47]. Among the 28 novel CpxR target genes with well annotated functions identified by bioinformatics based screening, we found two genes implicated in 1,2-propanediol utilization: *glhA* and *pocR* (Fig 8B) not being differently regulated under invasion inducing conditions. The *glhA* encodes a glycerol dehydrogenase with 1,2-propanediol as substrate and is a suppressor of *cpxA* in *S*. Typhimurium SL1344 rescuing *hilA* expression [47]. The *pocR* encodes an AraC type regulator modulating the cobalamine synthesis [79] and the 1,2-propanediol utilization (*pdu*) gene cluster [80–82]. EMSA verified the regulator PocR as a direct CpxR target (Fig 8C). In order to analyze the impact of the Cpx pathway on 1,2-propanediol utilization, we analyzed the fermentative growth on 1,2-propanediol according to an established protocol [24]. Of note, fermentative growth on 1,2-propanediol depends on dilute yeast extract (0.2%) as additional carbon source that cannot support anaerobic growth alone [24]. Fermentative growth on 1,2-propanediol was enhanced in a *cpxR* deletion strain (Fig 8D) supporting our assumption that the Cpx-system modulates through the regulator PocR the utilization of 1,2-propanediol as the electron donor for the alternative electron acceptor tetrathionate in anaerobic respiration.

## Conclusions

In the last decades the Cpx-envelope stress system of Gram-negative bacteria has been extensively studied and assigned to be crucially involved during the invasion of host cells [4, 5, 14, 83]. Previous studies revealed for the food-born pathogen *S*. Typhimurium that deletion of the sensor kinase CpxA represses expression of the SPI-1 T3SS regulator HilA and attenuates virulence but surprisingly, deletion of the response regulator CpxR had neither effect on *hilA* expression nor on virulence when the bacteria were grown under mild acid conditions [20]. Here, we confirmed this effect for *S*. Typhimurium SL1344 grown in SPI-1 inducing medium. Interestingly, we observed in addition that a *S*. Typhimurium strain lacking CpxA and CpxR showed no decrease in *hilA* expression. Notably, CpxA represents a bifunctional sensor kinase that is able not only to phosphorylate its signaling partner CpxR but also to hold the balance between active and inactive CpxR by dephosphorylation [6, 51]. In previous studies it has been reported that CpxR can be phosphorylated by additional phosphate donors as the acetyl-CoA pathway, independent of CpxA [49]. In this case, CpxR would remain in an activated phosphorylated state in the absence of CpxA and therefore CpxR would be able to inhibit *hilA* expression. Interestingly, similar effects were reported for the QseCB quorum sensing system involved in virulence regulation of uropathogenic *E*. *coli* (UPEC) [50]. Deletion of the sensor kinase QseC but not of the response regulator QseB significantly attenuated intracellular bacterial community formation and virulence [50]. Moreover, a *qseBC* deletion mutant behaved like wild-type strain contradicting the hypothesis that QseC may function through different response regulators but suggesting that dephosphorylation of QseB is required for virulence gene expression [50]. In agreement with this we suggest that the phosphatase activity of CpxA is important for *hilA* expression by inactivating the inhibitory effect of phosphorylated CpxR.

Here, we identified 1,2-propanediol degradation system (*pocR*) and the Tat-system (*tatABC*) which are involved in the expression of *hilA* to be under direct control of CpxR (Fig 8) [47, 65]. Interestingly, these two operons are functionally linked. Together they promote the anaerobic respiration of tetrathionate as electron acceptor on 1,2-propanediol as electron donor [24]. Anaerobic respiration on tetrathionate is a differential ability of Salmonella and therefore used in clinical diagnostic in a standard enrichment medium [74, 84, 85]. The *ttrABCRS* cluster responsible for tetrathionate respiration is located on SPI-2 [62, 74]. It was show that reactive oxygen species generated during inflammation, caused by the activity of both *S*. Typhimurium T3SSs [86], react with endogenous sulphur compounds to form tetrathionate which inhibits coliforms [77]. Consequently, tetrathionate respiration has been linked to Salmonella specific host adaptation that results in a growth advantage for *S*. Typhimurium over the competing microbiota in the lumen of the inflamed gut [77]. 1,2-Propanediol, the electron donor for the anaerobic respiration of tetrathionate, is a fermentative product from rhamnose and fucose catabolism, two sugars commonly present in the mammalian intestinal tract by e.g. plant cell walls [87]. The degradation of 1,2-propanediol is a complex process performed in an specific 1,2-propanediol utilization (Pdu) microcompartment in a Vit B12 dependent manner [79, 88]. *Salmonella* upregulates proteins for the utilization of 1,2-propanediol (pdu-cob cluster) under invasion-mimicking conditions [89]. Expression of the *pdu-cob* cluster is inhibited by PocR, induced by 1,2-propanediol and globally controlled by the ArcA and Crp systems [90]. The combined results indicate that deletion of CpxA results in active CpxR inducing expression (Fig 8). The PocR regulator inhibits expression of the *pdu-cob* cluster resulting in the accumulation of 1,2,-propanediol (PDO) and finally in *hilA* repression. Thus, our finding that the regulator for the degradation of 1,2,-propanediol PocR is under direct CpxR control suggests that the Cpx envelope stress system links metabolism with virulence regulation.

However, the exact mechanism of how the degradation of 1,2-propanediol and the Tat-system impacts the expression of *hilA* is not known. Transcription of *hilA* is controlled by a complex feed-forward loop including RtsA, HilC and HilD [46]. Environmental signals feed into this network and mediate *hilA* expression through post-transcriptional or post-translational control of *hilD*. One of the known post-transcriptional hilD regulators is the EnvZ/OmpR two-component system [46]. Recently, it was demonstrated that the CpxR target MzrA modulates the activity of EnvZ by direct protein-protein interaction in *E*. *coli* [91–93]. Accordingly, MzrA can be defined as an auxiliary protein that connects two two-component systems [94]. We identified the *mzrA* gene by global transcriptional analysis to be under global control of the Cpx envelope stress system. Of note, we also identified CpxR recognition motif in the *mzrA* promoter region in *S*. Typhimurium as described in *E*. *coli* suggesting that MzrA might play the mediator protein role between the CpxAR and the EnvZ/OmpR systems to modulate *hilA* expression (Fig 8 and S2 Fig) [92].

Identification of the Tat-system (*tatABCD*) as direct CpxR target in S. Typhimurium was unexpected since the *tatABCD* operon was described to be constitutively expressed in *E*. *coli* [72]. The Tat-system facilitates the export of cofactor-containing proteins across the cytoplasmic membrane [95]. In *E*. *coli*, the Tat-system consists of TatA, TatB, TatC, TatD and TatE proteins that are encoded on the *tatABCD* operon and *tatE* [96] respectively. The promoter region of the *tatE* gene that is thought to be a cryptic gene duplication of *tatA* does not consist of a CpxR recognition motif. Interestingly, a CpxR recognition motif could also not be identified upstream of the *tatABCD* operon in several other species including pathogenic *E*. *coli* strains, indicating that the regulated expression of *tatABCD* by CpxR is species-specific (Fig 6D). In line with this is the observation that for several pathogens the effects for tat mutants vary including growth rate, motility, biofilm formation, host colonization and virulence [65, 97–99]. As an example, tat mutants of Escherichia, Agrobacterium and Pseudomonas become non-motile [97, 100, 101] whereas no effect on Vibrio motility could be observed [98]. A *Salmonella* specific substrate of the Tat-system is the A subunit of the anaerobic tetrathionate reductase [74]. As stated above, anaerobic respiration on tetrathionate promotes *Salmonella* a growth advantage over the competing microbiota [77]. Moreover, two Tat-system dependent amidase (AmiA and AmiC) that confer resistance to cationic antimicrobial peptides (CAMPs) were shown to be under direct CpxR control [22]. Taking our experimental findings into consideration we conclude that the Cpx-dependent expression of the Tat-system is important in the ecology of Salmonella.

In addition to the disparate regulation of the Tat-system, we found a phenotypic variation of Cpx-dependent motility (*motABcheAW*). Motility of *S*. Typhimurium is under positive CpxR control, whereas E. coli motility is negatively regulated by the response regulator [37]. A phenotypic difference between E. coli and *S*. Typhimurium was first described for the resistance to the antimicrobial peptide polymyxin B, which is governed by the PmrA/PmrB system [102]. In contrast to *E*. *coli*, *S*. Typhimurium is resistant to polymyxin B under low Mg2+ condition [45]. In *S*. Typhimurium but not in *E*. *coli* dephosphorylation of the PmrA response regulator is prevented by the PmrD connector protein expressed by the PhoP/PhoQ system and responds to low extracellular Mg2+ [102, 103]. Moreover, many studies have underlined the difference in regulatory strategies between *Salmonella* and *Escherichia coli* [67–71]. Thus, our finding underlines the remarkable diversity in regulatory circuits between the closely related species *Escherichia* and *Salmonella* and exemplifies the assumption that disparate regulation of conserved genes has consequences for the ecological niches bacterial species can colonize [102].

Phenotypic differences among related bacteria are mainly assigned to species-specific genes. Here, we identified the genes for the SPI-2 regulator SsrB and the omptin PgtE as direct CpxR targets (Fig 8). SsrB is the response regulator of the SsrAB two-component system that activates the expression of the SPI-2 T3SS. In contrast to many other two-component systems expression of the sensor kinase SsrA and the response regulator SsrB is independently regulated from each other [73]. SsrB directly controls the expression of the SPI-2 T3SS and its effectors that provide Salmonella to survive in host cells [63, 104]. It has been reported that the SPI-2 T3SS is also expressed in the intestine independently of the invasion process but without substrate protein secretion [79] supporting a previous suggestion that the acidic pH environment typical for the interior of macrophages might be an essential trigger for the secretion of SPI-2 T3SS substrate proteins [104]. Consistent with this, we found no difference in the secretion pattern for the SPI-2 T3SS substrate protein SseB (S5 Fig). These data also support the observation that deletion of the Cpx-system results in only slight attenuated growth of *S*. Typhimurium in macrophages [21]. Accordingly, our results indicate that the Cpx-system might be involved in preparing Salmonella for its life in host cells.

**Fig 9:**
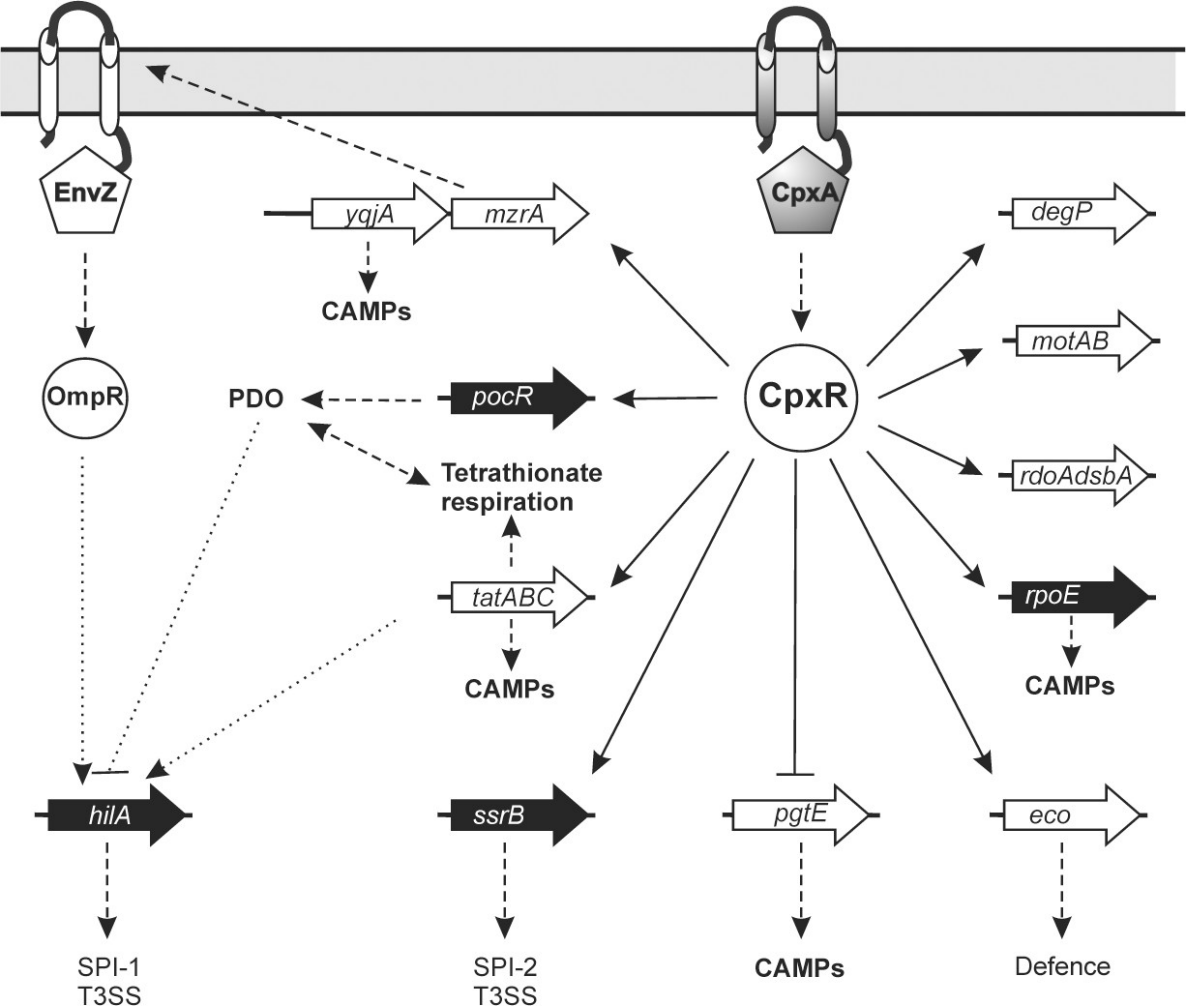
Model of the Cpx envelope stress network in *S*. Typhimurium. Genes are pictured within big arrows. Regulator genes are depicted within black arrows and the enzyme or transporters are depicted within white arrows. Thin arrows indicate a positive effect. Line with blunt ends note a negative effect. Solid lines represent direct transcriptional regulation. Regulation that is not known to be direct or indirect is represented by short-dashed lines. Long-dashed lines indicate post-translational effects. For clarity, the genes encoding HilD, HilC and RtsA are not shown. Abbreviations used are CAMPs, cationic antimicrobial peptides; PDO, 1,2-propaendiol; T3SS, type three secretion system. See text for details and references.

PgtE belongs to the omptin family of outer membrane β-barrel proteases that promote virulence associated functions of different pathogens [105]. Pla of Yersinia pestis is located on the virulence plasmid pPCP1, and advances the migration of the plaque bacteria through tissues [106]. SopA of Shigella flexneri is encoded on the plasmid pWR100 and is important for the intracellular mobility [105]. PgtE of Salmonella enterica promotes resistance towards CAMPs like polymyxin B and protamine [107]. CAMPs as part of the innate immune system are typically amphiphatic peptides of 12–45 residues length with wide variations in their sequences and secondary structures [108, 109]. Very recently, it has been proven that the Cpx-envelope stress system confers resistance to the CAMPs protamine, magainin-2 an melittin through regulated expression of the two Tat-system dependent amidase AmiA and AmiC [22]. These Tat-dependent amidases contribute to bacterial resistance to the CAMPs protamine, magainin 2 and melittin but not to the CAMPs HNP-1 and polymyxin B [22]. Recent studies in *S*. Typhimurium carrying *pgtE* deletions had 2-fold lower minimum inhibitory concentrations (MICs) to two CAMPs namely human LL-37 and its murine ortholog CRAMP [107], while *pgtE* overexpression increased the MIC by 8-fold (Band and Weiss 2015). Here, we demonstrate that the Cpx-system contributes also resistance to polymyxin B. Other CpxR targets that are known to confer resistance to CAMPs are the inner membrane protein of unknown function YqjA (S4 Fig) and extracytoplasmic sigma factor sE (RpoE) [110, 111]. As the Cpx-targets PgtE and YqjA which contributes to resistance towards protamine, our finding suggests the regulation of these two loci as the Tat-independent mechanism of Cpx pathway promoted protamine resistance. Collectively, our data point the Cpx-envelope stress system of *S*. Typhimurium a critical function during the early stage of infection. Starting with the competition with the invasion process (*hilA*, *pocR*, *tatABC*) to the preparation for the escape from the host cell immunity (*eco*, *ssrB*). Taking all findings together we are reporting here a comprehensive model depicting network regulated by Cpx pathway (Fig 9). Moreover, it seems that *S*. Typhimurium is able to tolerate a complete loss of the whole Cpx system better than an impaired incomplete system [21]. A recent quantitative proteome study identified a need for 10-fold excess of CpxP to inhibit the CpxRA two-component system [112]. The interaction of Cpx-system with other regulatory networks like small non-coding RNAs (sRNAs) has been reported [113].Thus, although the Cpx system appears not to be essential it still can be assigned to an important role in fine-tuning virulence. Further studies could shed light on if the Cpx system is involved in physical interaction with other regulatory networks like sRNAs. Together with the general ability of the Cpx system to sense a wide range of different external stimuli this work supports the notion that the Cpx system plays a central role in a complex network regulating the interaction between pathogen and host.

## Supporting information

S1 Fig CpxA-mediated transcription of *hilA* is specific for SPI-1 inducing conditions. Promoter-GFP fusion assay of SL1344 wild type (WT; white), *cpxA* (MA; red) strains transformed with a plasmid carrying the GFP fusion to the *hilA* promoter (pD.2E). Shown are the expression results (bars) for cultures grown under normal conditions (LB medium, pH 7.0) (A) or under SPI-1 inducing conditions (B). Fluorescent values were measured at values set at 485/ 540 nm, cell growth of bacterial cultures was monitored at OD_620_. Data represents means ± S.E.M. of at least biological triplicates (t-test).
(TIF)

S2 Fig EMSA analysis for *E.coli* tatA promoter. EMSA analysis to verify the species specificity shown by CpxR towards *E*. *coli tatA* promoter. Indicated promoter fragments (p) were incubated without or with increasing amounts of the purified and phosphorylated CpxR protein. The DNA-CpxR~P complexes were separated on 4% polyacrylamide gels. The corresponding molecular weights are indicated on the left. The positions of the promoter fragments are indicated (p), arrows show the higher molecular weight DNA-CpxR~P complexes. A fragment of the cpxP promoter region without the CpxR~P binding motif (-151 to -297) was used as negative control (c).
(TIF)

S3 Fig DNAse I footprinting assay for Forward strand of *ssrB*. DNase I footprinting analysis of the *ssrB* promoter performed with the probe for the coding strand with increasing amounts of 6His-CpxR~P protein (see Experimental Procedures). No significant binding of CpxR~P was observed.
(TIF)

S4 Fig DNA sequence comparison for *yqjA-mzrA* promoter region of *E. coli* and *S.* Typhimurium. Underlined nucleotide indicate the CpxR binding motif identified and confirmed in *E*. *coli* [114]. The starts of the coding sequences are highlighted in bold letters.
(TIF)

S5 Fig Effect of *cpxR* deletion on SseB secretion. *S*. Typhimurium wild type (SL1344), the *cpxR* mutant NOS01 and the complementation strain NOS01+pSSS11 were grown in MgM-MES medium. Hexadecane and cell pellet fractions were obtained as described above (SI Experimental procedures) and analyzed by immunoblotting. Given is a representative of three biological replicates.
(TIF)

S1 Table Global transcriptional analysis. Matrix of expression ratios between *S*. Typhimurium SL1344 and *S*. Typhimurium SL1344 *cpxA*::*kan* (SHS01) strains invasion inducing condition labeled with functional descriptions (provided as separate Excel spreadsheet). Raw data are available online (http://www.webarraydb.org).
(XLSX)

S2 Table CpxR~P controlled input operons used to construct the CpxR~P recognition weight matrix. (provided as separate Excel spreadsheet).
(XLSX)

S3 Table Putative CpxR~P target operons identified by *in silico* data analysis in *S.* Typhimurium LT2. (Provided as separate Excel spreadsheet).
(XLSX)
