# Correction: Contribution of the Cpx envelope stress system to metabolism and virulence regulation in *Salmonella enterica* serovar Typhimurium

**DOI:** 10.1371/journal.pone.0213297

**Published:** 2019-02-27

**Authors:** Sivaraman Subramaniam, Volker S. Müller, Nina A. Hering, Hans Mollenkopf, Daniel Becker, Ann Kathrin Heroven, Petra Dersch, Anne Pohlmann, Karsten Tedin, Steffen Porwollik, Michael McClelland, Thomas F. Meyer, Sabine Hunke

The following information is missing from the Funding statement: MM and SP were supported in part by USDA grants 2017-67017-26180 and 2017-67015-26085 and by NIH grant R01AI136520.
