# Supplementary figures and images for: Contribution of the Cpx envelope stress system to metabolism and virulence regulation in Salmonella enterica serovar Typhimurium
Source: PLoS One. 2019 Feb 4;14(2):e0211584. doi: 10.1371/journal.pone.0211584 (PMC6361445; doi:10.1371/journal.pone.0211584)

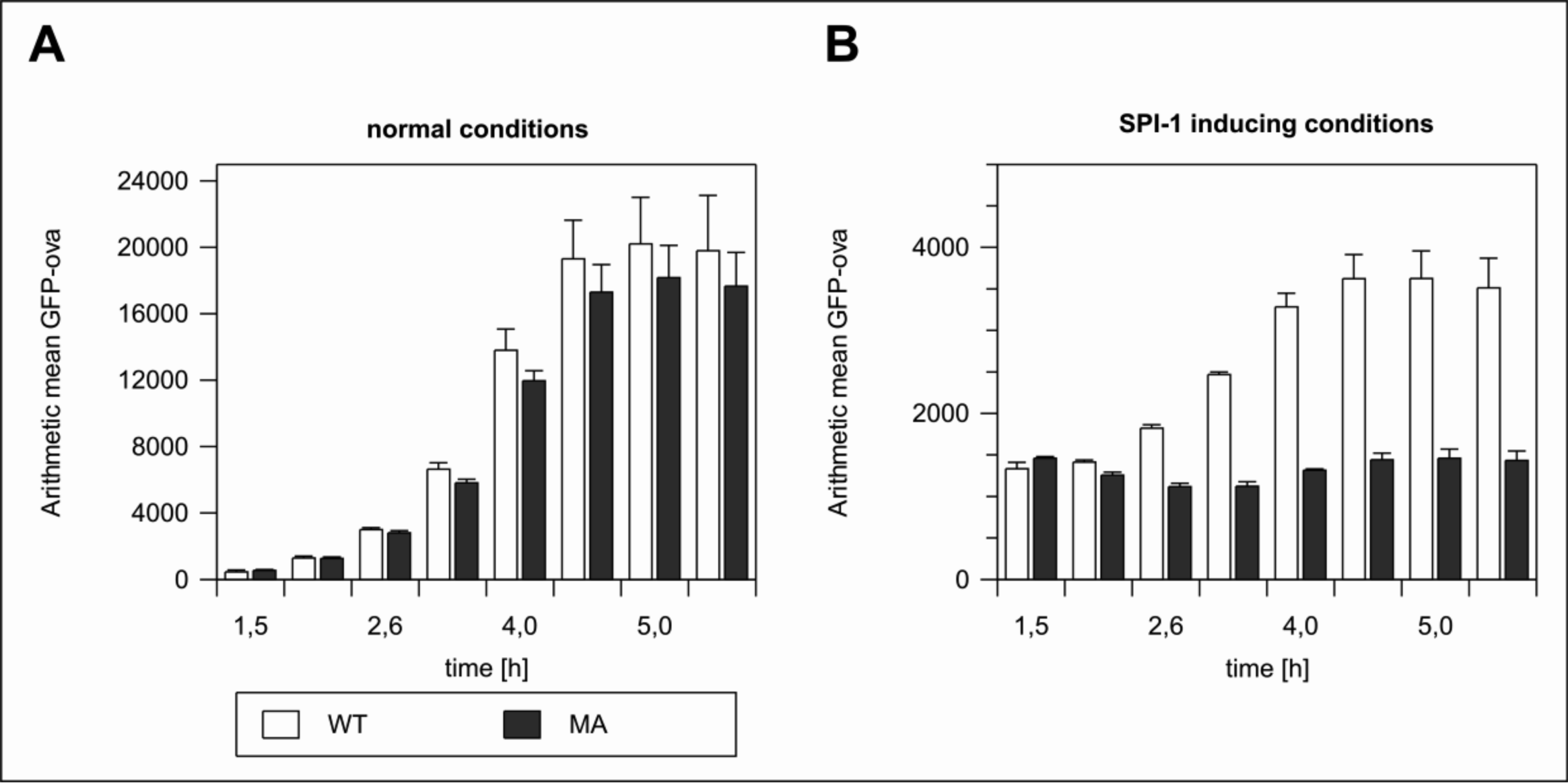

Supplement: S1 Fig — Promoter-GFP fusion assay of SL1344 wild type (WT; white), cpxA (MA; red) strains transformed with a plasmid carrying the GFP fusion to the hilA promoter (pD.2E). Shown are the expression results (bars) for cultures grown under normal conditions (LB medium, pH 7.0) (A) or under SPI-1 inducing conditions (B). Fluorescent values were measured at values set at 485/ 540 nm, cell growth of bacterial cultures was monitored at OD620. Data represents means ± S.E.M. of at least biological triplicates (t-test). (TIF) [file pone.0211584.s001.tif]

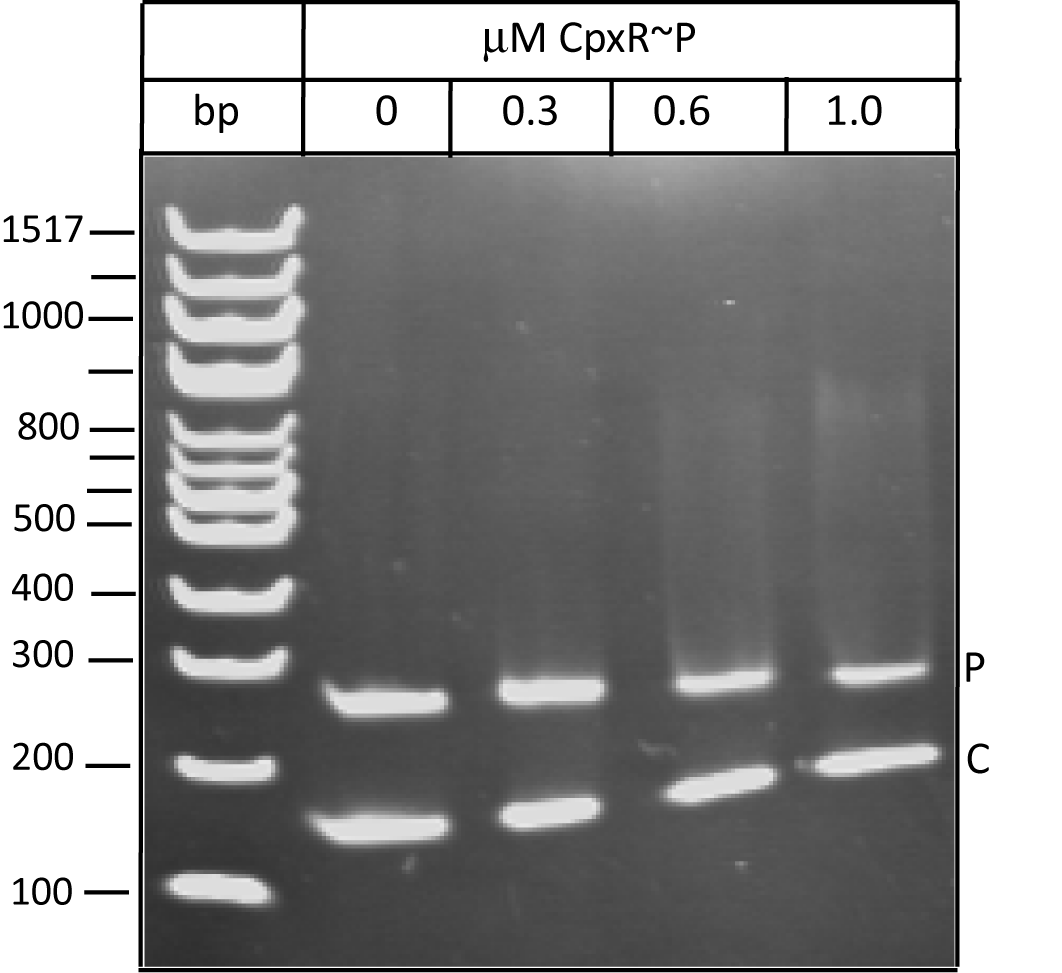

Supplement: S2 Fig — EMSA analysis to verify the species specificity shown by CpxR towards E. coli tatA promoter. Indicated promoter fragments (p) were incubated without or with increasing amounts of the purified and phosphorylated CpxR protein. The DNA-CpxR~P complexes were separated on 4% polyacrylamide gels. The corresponding molecular weights are indicated on the left. The positions of the promoter fragments are indicated (p), arrows show the higher molecular weight DNA-CpxR~P complexes. A fragment of the cpxP promoter region without the CpxR~P binding motif (-151 to -297) was used as negative control (c). (TIF) [file pone.0211584.s002.tif]

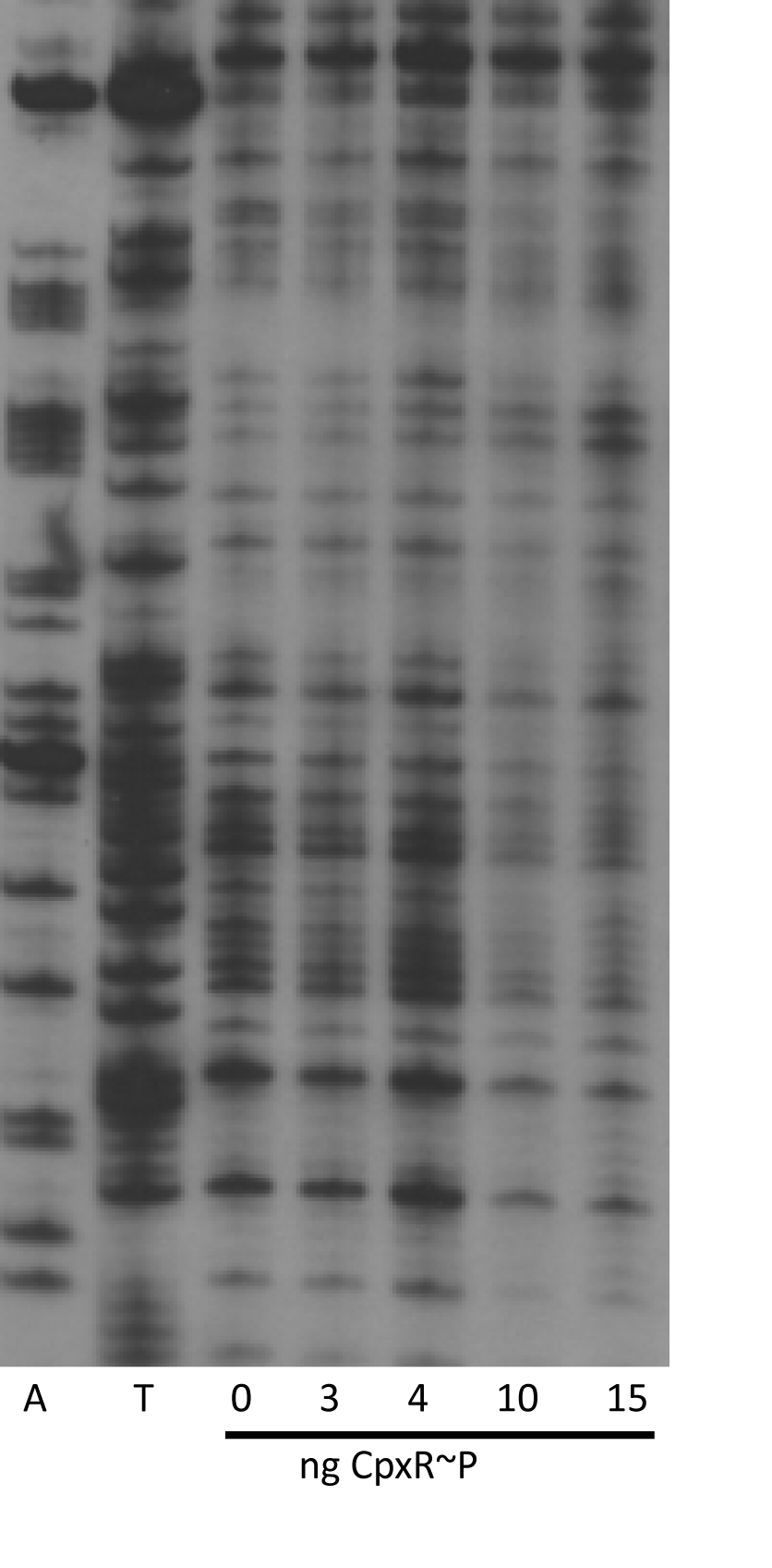

Supplement: S3 Fig — DNase I footprinting analysis of the ssrB promoter performed with the probe for the coding strand with increasing amounts of 6His-CpxR~P protein (see Experimental Procedures). No significant binding of CpxR~P was observed. (TIF) [file pone.0211584.s003.tif]

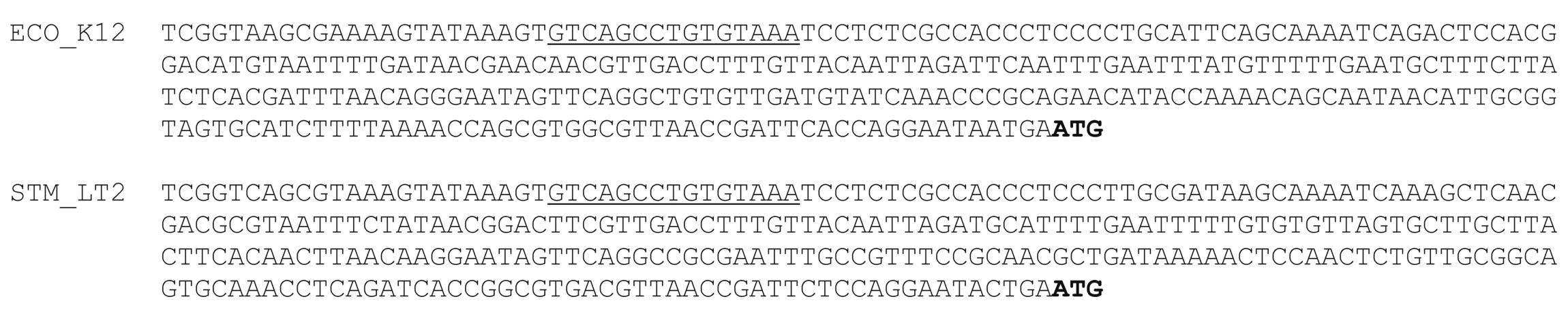

Supplement: S4 Fig — Underlined nucleotide indicate the CpxR binding motif identified and confirmed in E. coli [114]. The starts of the coding sequences are highlighted in bold letters. (TIF) [file pone.0211584.s004.tif]

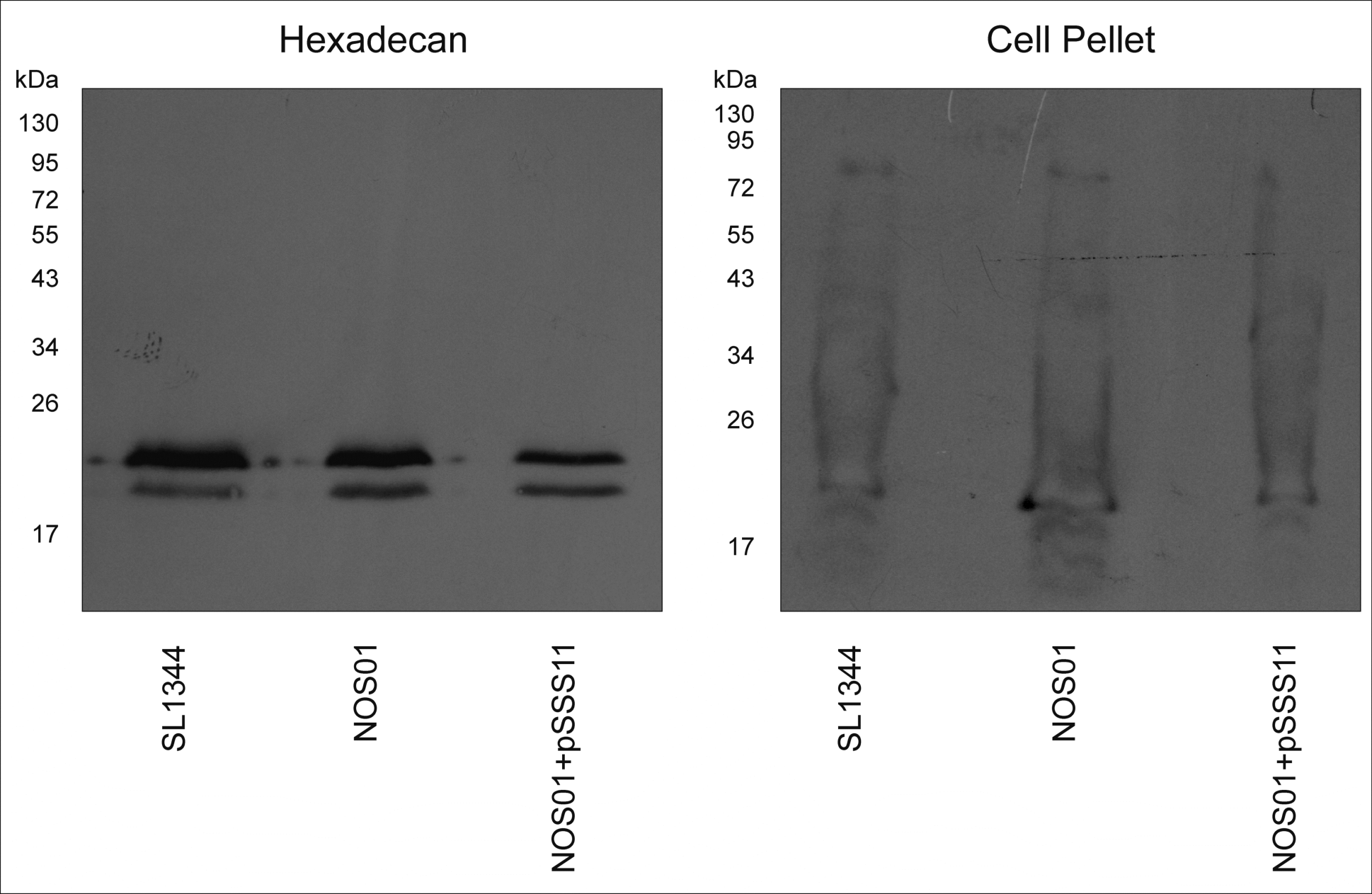

Supplement: S5 Fig — S. Typhimurium wild type (SL1344), the cpxR mutant NOS01 and the complementation strain NOS01+pSSS11 were grown in MgM-MES medium. Hexadecane and cell pellet fractions were obtained as described above (SI Experimental procedures) and analyzed by immunoblotting. Given is a representative of three biological replicates. (TIF) [file pone.0211584.s005.tif]
